# Mental health among the general population and healthcare workers during the COVID-19 pandemic: A meta-analysis of well-being and psychological distress prevalence

**DOI:** 10.1007/s12144-022-02913-6

**Published:** 2022-03-01

**Authors:** Ana Blasco-Belled, Claudia Tejada-Gallardo, Mònica Fatsini-Prats, Carles Alsinet

**Affiliations:** 1 University of Girona, Pujada de Sant Domènec, 9, 17004 Girona, Spain; 2 University of Lleida, Avinguda de l’Estudi General, 4, 25001 Lleida, Spain

**Keywords:** Mental health, Well-being, Psychological distress, COVID-19, Meta-analysis, Prevalence

## Abstract

The COVID-19 pandemic has constituted a global health crisis that has threatened the mental health of individuals worldwide. The present paper sought to systematically review and meta-analyze studies reporting the prevalence during the COVID-19 pandemic of well-being and psychological distress as defined by the dual-continua model, which includes (absence of) psychological distress and (presence of) well-being among the general population and healthcare workers. Systematic searches were conducted in various databases: PubMed, PsycINFO, Scopus, and Web of Science from inception until 6 December 2020. From a total of 158 studies (N = 880,352) included in the meta-analysis, only seven reported the prevalence of well-being. A random-effect model was used to estimate the pooled prevalence among the general population and healthcare workers on depression (25%; 31%), anxiety (27%; 31%), stress (35%; 32%), and well-being (52%; 45%), respectively. Sub-group analyses based on region, income, percentage of women, preparedness of country to respond to COVID-19, and economic vulnerabilities were conducted in order to examine sources of heterogeneity in psychological distress. Results revealed differences among the two groups and indicated that disparities in terms of preparedness to fight the pandemic can distinctly affect mental health in the general population and healthcare workers. Addressing mental health during and after a health crisis should be in the spotlight of the international and national public health agenda. Considering the protective role of well-being to minimize psychological symptoms, mental health policies during the COVID-19 should include strategies to combat the psychological consequences of the pandemic by promoting well-being practices.

**Supplementary Information:**

The online version contains supplementary material available at 10.1007/s12144-022-02913-6.

## Introduction

The COVID-19 pandemic has brought about a global health crisis with numerous and important consequences in different aspects of society. Political, economic, and medical efforts have understandably focused on the containment and eradication of the virus. Nevertheless, the derived psychological challenges, especially after the early stages of the pandemic, can further burden the global health system; as a result, mental health needs to become a priority (Gruber et al., 2021; Holmes et al., 2020). A wealth body of research has informed about the psychological consequences of the COVID-19 pandemic across the globe, using both cross-sectional and longitudinal data to monitor how changes on mental health unfold. Systematic reviews and meta-analysis have also evidenced the detrimental effects on mental health, but only considering the impact on psychological illness (e.g., Krishnamoorthy et al., 2020; Salari et al., 2020; Xiong et al., 2020). A review of the evidence during early 2021 reported that COVID-19 implied an early increase of psychological distress, as the levels of depression, anxiety, and stress escalated for months, but the majority of these measures generally returned to baseline levels by mid-2020 (Aknin et al., 2021).

Apart from the general population, a specific group that has faced this unprecedented situation is healthcare workers. Their duties were vital during the outbreak and course of the pandemic, taking risky decisions under extreme pressures, which puts them at greater risk of developing mental health problems like post-traumatic stress or affective disorders (Greenberg et al., 2020). Indeed, available evidence indicates that they reported increased psychological distress during this pandemic (Gruber et al., 2021; Planchuelo-Gómez et al., 2020). Hence, efforts to mitigate the psychological impact of healthcare workers are primary (Greenberg, 2020). To that end, research has called to urgently compile large-scale evidence about the psychological functioning of both general population and healthcare workers during and after the COVID-19 pandemic (Aknin et al., 2021; Holmes et al., 2020). Meta-analysis is a great tool to collect and analyze the existing data on the question at hand. But so far, and to our concern, all systematic and meta-analytic research has examined the impact on and unfolding of mental illness focusing on measures of depression, anxiety, stress, or sleep problems (e.g., Bueno-Notivol et al., 2021; Krishnamoorthy et al., 2020; Salari et al., 2020; Xiong et al., 2020). In contrast, no research has meta-analyzed the evidence of the impact on positive markers of mental health, such as subjective or psychological well-being. This gap highlights and aligns with the call to expand the psychological research to recognize the positive factors of mental health that facilitate better lives (Helliwell & Aknin, 2018).

The full scope of mental health entails more than the absence of psychopathology. As proposed by the dual-continua model of mental health (Keyes, 2005), scholars and practitioners should evaluate and intervene on indicators of psychological distress (e.g., stress, anxiety, or depression) and well-being (subjective, psychological, and social). Since well-being refers to how people feel about their life conditions (Ng & Fisher, 2013), understanding the changes on well-being can help guide decisions about how to best manage the psychosocial impact of the COVID-19 pandemic, assist in adopting more efficient measures to cease the spread of the virus and, more importantly, to develop strategies focused on the promotion of people’s quality of life (Aknin et al., 2021; De Neve et al., 2020). In general terms, research has found that the early phase of the pandemic was characterized by drops in positive emotions and greater experience of negative emotions. For instance, by comparing data between June 2019 and June 2020, Foa et al. (2020) reported a decline in positive affect and an increase in negative affect. Similarly, Zacher and Rudolph (2021) found in a German longitudinal study that subjective well-being decreased between March and May 2020, in comparison to the period between December 2019 and March 2020. But the impact on cognitive measures of subjective well-being (i.e., life satisfaction) seems to be more subtle. In their review, Aknin et al (2021) showed that life satisfaction remained unchanged (or even slightly increased) across multiple countries, although in some others it declined. The authors hypothesized that, because life satisfaction judgment entails a comparison between current life conditions with past or other people’s conditions, people might evaluate their life as better than expected given the pandemic circumstances.

Nevertheless, in view of the variability in the results about changes in well-being, systematization of the available literature including measures of psychological distress and well-being seems to be a tenable plan to understand the impact of the COVID-19 on mental health. According to this, the goal of the present research is to conduct a systematic review and meta-analysis of the prevalence of mental health following the dual-continua model of mental health to take into account indicators of psychological distress (i.e., depression, anxiety, and stress) and well-being (i.e., emotional, psychological, and social) among the general population and healthcare workers during the COVID-19 pandemic. Although no previous research covered this question by fully accounting on the positive and negative markers of mental health to base our hypothesis, we expect that the prevalence of psychological distress would be higher than the prevalence of well-being.

## Methodology

The preferred reporting items for systematic reviews and meta-analysis protocols (PRISMA-P) (Moher et al. 2015) was followed in the planning, implementation and reporting of the present meta-analysis. This study was pre-registered in PROSPERO, an international prospective register of systematic reviews, with the review number #CRD42020219372. The pre-registration information can be accessed at http://www.crd.york.ac.uk/prospero.

### Search Strategy

A systematic literature search was performed in the PsycINFO, PubMed, Scopus, and Web of Science databases. The reference list of previous reviews and meta-analyses was scrutinized to identify additional eligible studies. The search terms were: mental health, psychological well-being, social well-being, emotional well-being, subjective well-being, well-being, psychological distress, depression, anxiety, stress, general population, general public, healthcare workers, health professionals (last search carried out in December 2020). The search strings were combined according to the databases (See Table S1).

### Selection of Studies

The inclusion criteria for the selected studies were formulated in accordance with the PICOS approach and the studies were included based on the following criteria: studies (1) were cross-sectional designs reporting prevalence; (2) assessed indicators of either psychological distress or well-being; (3) were carried out since the outbreak announced by the WHO in January 2020; (4) included general population or healthcare workers; (5) included standardized and validated measures, and (6) were published in peer review journals. The exclusion criteria were the following: studies (1) included different subgroups of population (e.g., clinical population, other occupations than healthcare workers); (3) include individuals < 18 years old or samples of patients infected by COVID-19; (4) were not written in English or Spanish; (5) were study protocols grey literature or conference papers; (6) had incomplete or unidentified data.

### Data Extraction

Two authors (MFP and CTG) carried out the literature search and collected all the studies from the four databases. After the removal of duplicates, the retrieved articles were assessed for eligibility following a standardized procedure. First, the title, abstract and keywords were independently screened. Secondly, the full text was assessed according to the inclusion and exclusion criteria. In cases where the inclusion or exclusion of a study required further discussion, a third researcher (ABB) examined the disagreements so that a consensus could be reached. Data extraction templates were used to extract all the data from the included studies, which comprised the source of the study (author, publication, and date), participants (general population or healthcare workers), study design (cross-sectional studies) and outcomes (indicators of well-being and psychological distress).

### Quality Assessment

All the selected studies were subjected to critical appraisal for their methodological quality using the Joanna Briggs Institute (JBI) Critical Appraisal Checklist for Studies Reporting Prevalence Data (Munn et al., 2015) by two independent reviewers (MFP and CTG). The JBI checklist for prevalence studies evaluates nine domains: (1) sample frame adequacy, (2) participants sampling, (3) sample size, (4) participants and setting description, (5) data analysis coverage, (6) diagnostic methods, (7) the reliability and standardization of measurements, (8) statistical analysis adequacy, and (9) the response rate management. Reviewers rated each study using the options “yes”, “no” and “unclear”, which were taken as “low”, “high” and “unclear” risk of bias, respectively. The option “not applicable” was also available for each statement. For the total score, the number of “yes” answers was summed, in which a higher number of “yes” denoted less risk of bias. Any disagreements were resolved by discussion. The choice to include or exclude a study was based on the individual assessment of each study with an overall appraisal (either inclusion or exclusion).

### Statistical Analysis

Estimated prevalence rates for well-being and psychological distress were extracted from the studies and pooled prevalence estimates were calculated using the MetaXL version 5.3 (Barendregt & Doi, 2011) implementing the Freeman-Tukey double arcsine transformation and the normalization of prevalence before pooling the effect estimates. A random-effects model was selected to calculate the pooled prevalence, which allows for between-study variation by assuming that each study prevalence are normally distributed (Borenstein et al., 2010).

The Cochrane’s Q and I^2^ tests were applied to quantify the degree of inconsistency and heterogeneity. The Q test estimates whether there is heterogeneity in the study, whereas the I^2^ test indicates the percentage of variance that can be attributed to such heterogeneity, with percentages above 60–70% revealing substantial heterogeneity (Higgins & Thompson, 2002). Because we anticipate high heterogeneity, sub-group analyses were planned. We used several criteria to inspect the source of potential differences: WHO region; World Bank income group; % of women included in the study; human development index (HDI; Human Development Report Office, 2019); and the global index of COVID-19 preparedness of countries based on the number of hospital beds, physicians, and nurses per 10,000 citizens, and the countries current health expenditure (Kovacevic & Jahic, 2020).

To identify potential threats of unpublished studies, we evaluated indicators of publication bias through funnel plots and the Egger and Begg tests (Sterne et al., 2008). Publication bias were checked graphically be means of funnel plots, which represented the effect size of the different outcomes by plotting the overall mean effect size against study size. A symmetric distribution around the effect size denotes no publication bias, while an accumulation of the distribution on one side or the other of the effect size indicates publication bias.

## Results

### Study Selection

**Fig. 1:**
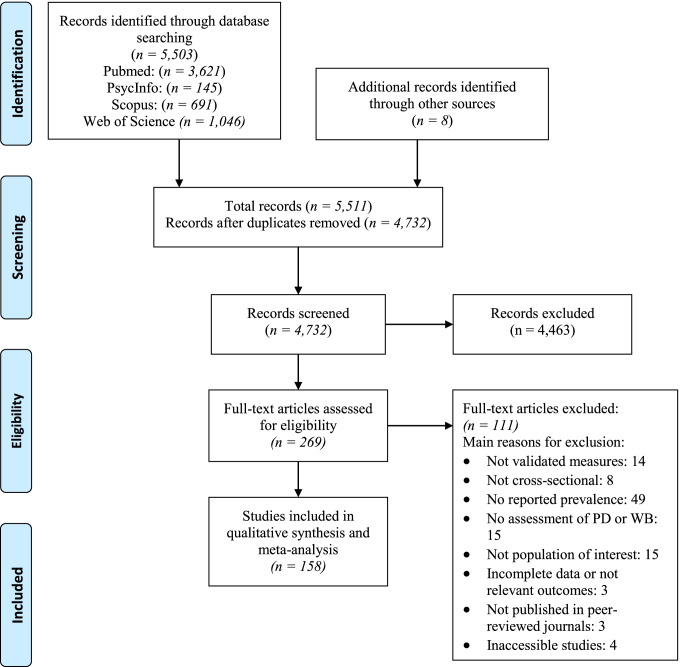
Study selection flow diagram

A total of 5,511 studies were found in the electronic databases: 3,621 from PubMed, 145 from PsycINFO, 691 from Scopus and 1,046 from Web of Science. Eight studies were also selected from other sources. After removal of duplicates, 4,732 studies remained for the title and abstract screening. In the next phase, 4,463 studies were discarded because they did not meet the inclusion criteria and 269 studies remained to review the full text. Of these, a total of 158 studies were included in the final meta-analysis (see Fig. 1 and Table S2). The main reasons of exclusions were that studies did not report the prevalence, sampled populations out of the scope of this research (e.g., clinical), and used non-standardized or validated measures.

### Description of Studies

A total of 158 studies involving 880,352 individuals from 41 countries were included in the meta-analysis, with the majority being conducted in China (33%). Of note, one study (Alzueta, 2020) involved 59 different nationalities (see Table S3). The age of participants ranged from 18 to 87 (M = 38.6) years old. A total of 344 effect sizes were calculated: 139 for depression, 155 for anxiety, 50 for stress, and seven for well-being (Table 1). The studies used a variety of measures to assess psychological distress such as the Depression, Anxiety, and Stress Scale (DASS-21; Lovibond & Lovibond, 1995), the Generalized Anxiety Disorder (GAD-7; Spitzer et al., 2006) or the Patient Health Questionnaire (PHQ-9; Kroenke et al., 2001). Regarding well-being, studies employed measures of subjective well-being like the WHO Well-Being Index 5 (WHO-5; Topp et al., 2015), and a combination of subjective and psychological well-being like the Warwick-Edinburgh Mental Well-being Scale (WEMWBS; Tennant et al., 2007) or the Psychological General Well-being Index (PGWB; Grossi & Compare, 2014). Due to the limited studies, we decided to meta-analyze the well-being studies altogether without distinctions between subjective or psychological well-being. The main characteristics of the included studies in the meta-analysis are presented in Table S3.

**Table 1:** Summary of studies reporting the prevalence of outcomes

Outcome	Population	Number of studies	Prevalence (95% CI)	Z-test (*p*)	I^2^	Q	Forest plot
Depression							
	General population	79	25% (0.23—0.27)	0.39 (.35)	100%	p = 0.00	Figure 1
	Healthcare workers	60	31% (0.26—0.35)		99%	p = 0.00	Figure 2
Anxiety							
	General population	84	27% (0.23—0.30)	0.89 (.53)	100%	p = 0.00	Figure 3
	Healthcare workers	66	31% (0.27—0.36)		99%	p = 0.00	Figure 4
Stress							
	General population	26	35% (0.28—0.41)	0.20 (.65)	100%	p = 0.00	Figure 5
	Healthcare workers	24	32% (0.23—0.42)		100%	p = 0.00	Figure 6
Well-being							
	General Population	4	52% (0.32—0.72)	0.98 (.32)	99%	p = 0.00	Figure 7
	Healthcare workers	3	45% (0.17—0.75)		99%	p = 0.00	Figure 8

### Quality Assessment

Table S4 shows the final rates of quality assessment for each study. Following the JBI checklist for prevalence, 145 studies presented scores of six or more, considering these studies as low risk of bias, 11 studies scored five, which represented some concerns, and only two studies scored four or less (Song, 2020a; Liu, 2020c), which represented high risk of bias. The domain of sample frame adequacy showed the lowest quality scores, followed by the response rate management. This means that the included studies (1) did not address appropriately the target population, presumably because they included non-representative samples or did not provide knowledge about the broader characteristics of the population, and (2) did not discuss information about how response rates were handled.

### Prevalence of Outcomes

The pooled estimated prevalence was 25% (95% CI 0.23—0.27) for depression among the general population and 31% (95% CI 0.26—0.35) among healthcare workers; 27% (95% CI 0.23—0.30) for anxiety among the general population and 31% (95% CI 0.27—0.36) among healthcare workers; 35% (95% CI 0.28—0.41) for stress among the general population and 32% (95% CI 0.23—0.42) among healthcare workers; and 52% (95% CI 0.32—0.72) for well-being among the general population and 45% (95% CI 0.17—0.75) among healthcare workers during the pandemic.^1^ Contrary to our expectations, the prevalence of well-being was higher than that of psychological distress among the two groups. In all cases, substantial degree of heterogeneity was found (I^2^ = 99—100% and p = 0.000 in all analysis) and therefore sub-group analysis were conducted for depression, anxiety, and stress. Due to the small number of studies in well-being, sub-group analyses were not performed.

### Sub-group Analysis

Table 2 shows the sub-group analysis for each of the psychological distress indicators. The prevalence of psychological distress among the general population was higher in regions of America, Europe and South-East Asia for anxiety and stress (p < 0.05); with upper-middle income for anxiety (p < 0.05), depression and stress (p < 0.001); in studies reporting > 60% of women for depression (p < 0.001); with medium–high human development for anxiety and stress (p < 0.01); with medium–low and medium–high percentage of physicians; with medium–low percentage of nurses per 10,000 citizens for anxiety (p < 0.05); with medium–low number of beds per 10,000 citizens for stress (p < 0.001); and with low and high health expenditure for anxiety and stress (p < 0.01).

**Table 2:** Sub-group analysis of the included studies on depression, anxiety, and stress

	General population				Healthcare workers			
	N total studies	Prevalence depression (95% CI)	Prevalence anxiety (95% CI)	Prevalence stress (95% CI)	N total studies	Prevalence depression (95% CI)	Prevalence anxiety (95% CI)	Prevalence stress (95% CI)
Region*								
Africa	2	-	-	-	2	-	-	-
Americas	22	.26 (.00, .61)	.30 (.12, .50)	.44 (.05, .85)	8	.36 (.06, .69)	.28 (.09, .49)	-
South-east Asia	11	.34 (.12, .59)	.21 (.14, .27)	.33 (.00, .90)	14	.11 (.07, .15)	.17 (.11, .24)	.35 (.00, .86)
European	75	.21 (.16, .25)	.22 (.15, .28)	.31 (.18, .45)	33	.49 (.32, .67)	.53 (.36, .70)	.46 (.22, .70)
Eastern Medit	9	.32 (.28, .36)	.11 (.00, .36)	-	20	.33 (.25, .40)	.30 (.21, .39)	.27 (.20, .34)
Western Pacific	65	.21 (.12, .30)	.18 (.09, .28)	.26 (.01, .60)	71	.29 (.21, .36)	.27 (.20, .34)	.31 (.10, .54)
* X*^*2*^ (*p*)		7.48 (.11)	11.65 (.02)	7.77(.05)		34.94(< .001)	.33 (< .001)	8.85 (.03)
Income								
Lower-middle	7	.27 (.13, .41)	.33 (.23, .4)	.24 (.00, .60)	13	.22 (.12, .33)	.27 (.19, .36)	.33 (.09, .59)
Upper-middle	10	.51 (.18, .84)	.38 (.16,.62)	.55 (.26, .83)	7	.38 (.16, .61)	.38 (.14,.64)	.48 (.17, .80)
High	78	.23 (.15, .31)	.22 (.08,.38)	.27 (.15, .40)	54	.32 (.24, .39)	.33 (.24,.43)	.32 (.15, .50)
* X*^*2*^ (*p*)		20.54 (< .001)	6.26 (.04)	25.59 (< .001)		6.15 (.04)	2.76 (.25)	6.84 (.03)
Females								
< 60%	29	.22 (.13, .32)	.22 (.03, .44)	.27 (.00, .87)	24	.35 (.22, .49)	.35 (.24, .45)	.44 (.17, .73)
> 60%	62	.27 (.21, .33)	.25 (.09, .38)	.33 (.23, .44)	49	.30 (.23, .38)	.30 (.22, .38)	.32 (.18, .48)
*X*^*2*^ (*p*)		21.96(< .001)	.25 (.62)	.86 (.35)		.34 (.56)	2.76 (.25)	6.84 (.03)
Human development								
Low	1	-	-	-	5	.22 (.02, .48)	-	-
Medium–low	4	.34 (.12, .59)	.25 (.17, .34)	.33 (.00, .90)	6	.24 (.08, .41)	.29 (.10, .50)	.15 (.00, .35)
Medium	32	.21 (.10, .33)	.14 (.04, .26)	.30 (.00, .69)	35	.29 (.21, .36)	.23 (.15, .32)	.34 (.10, .61)
Medium–high	9	.31 (.26, .36)	.37 (.25, .50)	.48 (.14, .82)	11	.39 (.24, .54)	.41 (.30, .52)	.41 (.20, .63)
High	47	.24 (.12, .37)	.27 (.13, .43)	.28 (.17, .39)	19	.41 (.25, .57)	.43 (.26, .61)	.25 (.07, .46)
* X*^*2*^ (*p*)		5.40 (.14)	13.95 (.003)	10.80 (.01)		13.93 (.007)	12.30 (.006)	18.59 (< .001)
Preparedness – Physicians								
Low	0	-	-	-	0	-	-	-
Medium–low	5	.33 (.14, .54)	.28 (.17, .39)	.33 (.00, .90)	8	.17 (.07, .28)	.23 (.12, .35)	.29 (.06, .57)
Medium	32	.19 (.11, .29)	.14 (.04, .25)	.27 (.00, .88)	44	.31 (.23, .38)	.29 (.22, .35)	.36 (.17, .56)
Medium–high	22	.26 (.04, .52)	.29 (.13, .47)	.39 (.06, .75)	12	.31 (.18, .44)	.30 (.17, .45)	.43 (.02, .88)
High	33	.22 (.16, .29)	.22 (.09, .38)	.30 (.17, .45)	11	.46 (.21, .71)	.52 (.25, .78)	.24 (.18, .31)
* X*^*2*^ (*p*)		5.87 (.12)	7.99 (.04)	3.60 (.31)		19.58 (< .001)	21.77 (< .001)	9.32 (.03)
Preparedness – Nurses								
Low	2	.26 (.12, .42)	.31 (.26, .36)	-	3	-	.24 (.08, .42)	-
Medium–low	3	-	.42 (.32, .53)	-	4	.37 (.28, .47)	.42 (.26, .58)	.24 (.15, .35)
Medium	35	.19 (.11, .29)	.13 (.93, .25)	.26 (.00, .81)	45	.29 (.21. .36)	.27 (.21, .34)	.35 (.13, .59)
Medium–high	25	.20 (.14, .27)	.22 (.15, .33)	.31 (.18, .45)	14	.43 (.25, .61)	.48 (.31, .66)	.30 (.11, .49)
High	28	.26 (.08, .47)	.29 (.09, .38)	.37 (.15, .61)	9	.30 (.15, .45)	.26 (.14, .39)	.27 (.00, .77)
* X*^*2*^ (*p*)		2.43 (.49)	23.39 (< .001)	2.82 (.24)		5.68 (.13)	21.18 (< .001)	3.21 (.36)
Preparedness – Beds								
Low	5	.27 (.13, .41)	.31 (.23, .40)	.24 (.00, .60)	8	.19 (.08, .32)	.27 (.15, .41)	.36 (.00, .85)
Medium–low	5	.28 (.14, .43)	.30 (.13, .49)	.66 (.20, .90)	6	.26 (.15, .38)	.26 (.13, .39)	.24 (.16, .31)
Medium	15	.37 (.19, .56)	.30 (.17, .43)	.48 (.09, .88)	8	.38 (.13, .65)	.34 (.22, .48)	.35 (.05, .69)
Medium–high	65	.22 (.14, .31)	.22 (.07, .40)	.27 (.15, .40)	48	.32 (.24, .40)	.31 (.22, .40)	.35 (.15, .57)
High	2	.22 (.05, .43)	.17 (.01, .38)	-	4	.35 (.19, .52)	.42 (.14, .70)	.54 (.00, .99)
* X*^*2*^ (*p*)		7.62 (.11)	8.00 (.09)	47.81 (< .001)		10.95 (.03)	7.63 (.11)	20.07 (< .001)
Preparedness – Health expenditure								
Low	7	.30 (.19, .41)	.31 (.20, .43)	.41 (.01, .85)	9	.17 (.08, .27)	.25 (.15, .36)	.26 (.04, .52)
Medium–low	31	.19 (.11, .29)	.13 (.04, .23)	.25 (.00, .76)	41	.30 (.23, .38)	.28 (.21, .34)	.36 (.15, .57)
Medium	4	.32 (.17, .49)	.25 (.19, .31)	-	5	.36 (.03, .73)	.33 (.15, .53)	-
Medium–high	22	.19 (.14, .25)	.24 (.15, .34)	.30 (.17, .43)	12	.45 (.23, .68)	.51 (.28, .75)	.24 (.12, .37)
High	29	.26 (.07, .47)	.29 (.12, .47)	.42 (.19, .67)	8	.34 (.20, .48)	.35 (.16, .56)	.66 (.48, .84)
* X*^*2*^ (*p*)		7.79 (.10)	10.58 (.03)	9.25 (.03)		19.05 (< .001)	18.04 (.001)	47.88 (< .001)

*Alzueta et al. (2020) was not included in any sub-group analysis because it included 59 different countries and the data could not be introduced

In the case of healthcare workers, the prevalence of psychological distress was higher in studies from regions of Europe for stress (p < 0.01) anxiety and stress (p < 0.001); with upper-middle income for depression and stress (p < 0.05); in studies that involved < 60% of women for stress (p < 0.05); with high human development for depression (p < 0.01) and medium–high human development for anxiety (p < 0.01) and stress (p < 0.001); with medium to medium–high physicians per 10,000 citizens for depression, anxiety (p < 0.001) and stress (p < 0.05); with medium–high nurses for anxiety (p < 0.001); with high number of beds for stress (p < 0.001); and with medium–high to high health expenditure for the three indicators of psychological distress (p < 0.001).

The results thus reveal differences between the general population and healthcare workers. Overall, healthcare workers from countries with higher rates of human development, physicians and health expenditure reported higher prevalence of depression, anxiety and stress, whereas the general population showed higher psychological distress in countries with less resources, such as medium–low nurses (for anxiety) and beds (for stress), and health expenditure (for anxiety and stress). Of note, in all sub-group analysis except for anxiety in healthcare workers, upper-middle income countries reported significantly higher rates than those with lower-middle income. Psychological distress was higher for healthcare workers than for the general population in European regions, while in regions of America and South-east Asia the prevalence was generally higher for the general population. Studies with greater rates of women in the general population displayed higher depression, whereas studies involving less rates of women in healthcare workers reported more stress.

## Discussion

To understand the full scope of mental health within the framework of this global health crisis, research is called to urgently provide clear and comprehensive evidence of the psychological consequences of COVID-19 so to help guide political, social, and economic decisions. To that end, the present meta-analysis is the first to measure the prevalence of mental health from the dual-continua model during the COVID-19 pandemic, which not only takes into account markers of psychopathology, but also of well-being as two different but related dimensions. Our meta-analysis included a total of 158 studies (*n* = 880,352) that assessed the prevalence of depression, anxiety, stress, and subjective/psychological well-being, comparing the results among the general population and healthcare workers.

The pooled prevalence in all markers was similar among the two groups, with healthcare workers reporting slightly higher rates of anxiety and depression and lower well-being than the general population. Similar to recent reviews (Nochaiwong et al., 2021), stress was the outcome with the highest prevalence, followed by anxiety and depression. Compared to recent reports on the global prevalence of mental disorders (Dattani et al., 2021; Steel et al., 2014; World Health Organization, 2017), our results showed that the rates of depression, anxiety, and stress have increased between 2—8 times since the outbreak of COVID-19.

But the present study extends previous meta-analytic findings because, for the first time, the prevalence of positive indicators of mental health was meta-analyzed. Results showed that the prevalence of well-being was higher than that of the psychopathological indicators, suggesting that protective components of mental health were present during this pandemic. Unlike psychological distress data, no meta-analytic evidence exists, to the best of our knowledge, about the prevalence of well-being prior to COVID-19. Yet, some studies attributed estimates between 20%—50% of flourishing^2^ in general adult populations (Keyes et al., 2008; Petrillo et al., 2014; Yin et al., 2013). This is important from the dualistic model of mental health, since an enhanced well-being can protect and offer treatment approaches to handle the negative effects of psychological distress (Fava et al., 2017; Johnson & Wood, 2017). The fact that only seven of the included studies assessed the prevalence of well-being reflects the current inequal interest in monitoring the positive factors of mental health within the psychological research. Generally, some of the included studies that measured well-being did not provide the prevalence, but rather offered other estimates, and they could not be included in the meta-analysis. Because clinical psychology and psychiatry rely on diagnostic criteria, these disciplines are used to identify specific cut-off scores of malfunctioning. Contrarily, in well-being research, estimates are typically described as continuum variables (e.g., on a 7-point scale) and are used as outcomes or correlates of other psychological phenomena. If psychological research is to capture the full scope of mental health, the first step is to provide and use measures to detect (also) the levels of well-being.

These findings align with the call to recognize aspects of well-being that go beyond the absence of psychopathology to fully tap into individuals’ mental health (Helliwell & Aknin, 2018; Johnson & Wood, 2017). The path toward such goal requires systematic collaborations across different scientific disciplines, involving clinical or developmental psychology, and sociology, neuroscience, or economics to draw firm insights about the unfolding of mental health from a broader perspective. Only with interdisciplinarity it will be possible to provide and design better public policies to face the prevailing effects of this pandemic and ensure mental health, but also to prepare people for a future global health challenge.

### Differences Among the General Population and Healthcare Workers

As expected, we found high heterogeneity across studies. Therefore, it is important to understanding the sources of variability in the results. There were differences between the two groups, notably in relation to the number of physicians, nurses, beds and healthcare expenditure. For example, the general population experienced higher anxiety in countries where the number of physicians was medium–low to -high, whereas healthcare workers experienced higher depression, anxiety and stress in countries where the number of physicians was high and medium–high. In general, the results suggest that the psychological impact of COVID-19 has been greater to healthcare workers than to the general population. We presume that the workload and pressure supported by healthcare workers during this pandemic have affected more in-depth their mental health compared to the general population, as depression and anxiety showed a particularly higher prevalence when the number of physicians was high. Interestingly, the prevalence of stress in healthcare workers increased when the rates of physicians were medium–high. This might be explained by the role that stress has in coping with transitionary life challenges (Gutowski et al., 2018). The number of nurses influenced differently the prevalence of anxiety among the two groups: while the general population reported higher anxiety when the rates of nurses was medium–low, healthcare workers reported more anxiety when the rates were medium–low to -high. These results suggest that the psychological consequences of COVID-19 in the general population might be more accentuated when there were less nurses than physicians. By contrast, mental health in healthcare workers was more affected when there were less physicians than nurses.

Another noted difference regards to the number of beds per 10,000 citizens. The general population reported more stress in countries with medium–low number of beds; by contrast, healthcare workers reported more stress in countries with high number of beds. Similarly, healthcare expenditure exerted a factor of difference among the two groups. The general population indicated higher anxiety and stress in countries with low and high investment. The healthcare workers, by contrast, showed more depression and anxiety when the investment was medium–high, and higher stress when the investment was high. Taken together, the results might reveal that psychological distress was greater among healthcare workers than the general population when more infrastructure and personnel resources were available, such as the number of physicians, beds or healthcare expenditure. Conversely, psychological distress in the general population resulted more affected when the amount of those same resources was minor.

Even though there was not much difference between the groups, this finding should be interpreted carefully. A reasonable explanation refers to the demanding expectations that healthcare workers face when they have access to larger resources to attend the population. As such, managing healthcare assets during this pandemic (which at some point became limited and crucial) may have induced healthcare workers a pressure to save as much lives as possible. In countries with lower personnel and worse equipped, the expectations may have been more limited and therefore the stress to manage the resources could have been not as demanding. The challenging task of organizing health teams to continuously learn new (and rapidly changing) protocols, the uncertainty of whether their performance was correct, or the pressure to make moral decisions can also explain the poorer mental health of healthcare workers.

In the general population, literature showed that aspects such as the lockdown and problematic access to healthcare or housing have significantly disadvantaged people from countries with lower HDI (Smith, 2020), which can be related to higher risk of psychological distress. Another explanation can be associated with the subjectivity of outcome responses (Khan et al., 2021) or other factors inherent to the workplace (e.g., communication or social support; Spoorthy et al., 2020).

### Limitations

Several limitations need to be acknowledged. First, a high degree of heterogeneity was found across the included studies. Even though we performed sub-group analyses, other characteristics of the studies were left behind, such as the study design or the moment of publication (initial or advanced stages of the pandemic). Nevertheless, research warns about the use of the I^2^ statistic because it depends on the study precision and the number of included studies, especially if the final sample size is large (Steel et al., 2014). Other factors can be considered as source of difference among studies, such as age and the severity of COVID-19, which may imply more psychological distress in countries that face more challenging and risky conditions. Second, we focused on cross-sectional studies and therefore the unfolding of mental health was not examined. A longitudinal meta-analysis would assist in monitoring the evolution of mental health development during different stages of the pandemic. Similarly, only studies published in academic journals were eligible, and non-published work (e.g., pre-prints) may have smaller effects. Third, a variety of well-being definitions and non-validated measures were found across studies. Incorporating validated measures of well-being that encompass emotional, psychological, and social dimensions of well-being would help evaluate more comprehensibly well-being. Likewise, the number of included studies assessing well-being felt short compared to those measuring psychological distress, which might pose a threat to generalize the results. In this sense, the number of included studies in the sub-group analysis differed and this could potentially affect the results, therefore readers should carefully take into consideration the generalization of these particular results. Fourth, well-being was not always the primary outcome of the included studies, which highlights the importance of including well-being measures when studying the mental health of population. Fifth, some studies included participants with pre-existing mental health issues, which could affect the results. It would be interesting to introduce pre-screening measures when studying non-clinical samples. Sixth, the studies included are quantitative in nature, limiting the scientific approach of the theme. Future studies on the topic would also enrich from qualitative data. Additionally, most of the evidence included in this study is drawn essentially from western, educated, industrialized, rich, and democratic nations (WEIRD; Henrich et al., 2010). Hence, the present findings must be interpreted with caution because the prevalence of mental health could be influenced by other factors, such as individual differences, education, or culture. Also, the response of countries, and subsequently the effects on mental health, vary in regions with lower-middle income, HDI or health resources (Hale et al., 2021; Kola et al., 2021). This limitation raises important opportunities for future research.

### Implications

Public health and governments need to advocate for an increased awareness of psychological distress, but also well-being requires a special attention to adapt to and address challenging and uncertain events like the COVID-19 pandemic. It is important to provide interventions focused on easily-implemented and routine-based practices in order to promote mental health among the general population and healthcare workers. With the new demands that psychological science face in relation to clinical assessment and treatment, research is called to integrate positive and negative aspects of well-being to bring useful resources to practitioners (Wood & Tarrier, 2010). Although the efficacy of positive psychology interventions in enhancing mental health has been scientifically demonstrated in different populations and contexts (Hendriks et al., 2019; Sin & Lyubomirsky, 2009; Tejada-Gallardo et al., 2020), its application in health settings is rare. The evidence invites to consider the potential benefits of implementing positive intervention to healthcare workers. For example, declines in subjective well-being were related to stress appraisals and coping strategies during early stages of the COVID-19 pandemic (Zacher & Rudolph, 2021). The authors showed that appraising the crisis as less threatening, less central, and more challenging and controllable; training and developing certain problem-focused, emotion-focused, and socially supported strategies; and disengaging from avoidant-coping strategies proved helpful to enhance well-being.

Another example is that resilience strategies in healthcare workers can be implemented to help minimize the psychological consequences of this pandemic (Heath et al., 2020). From a broader perspective, the effects of public policies on well-being beyond traditional economic metrics, opens new possibilities to list priorities and achieve societal progress (Cylus et al., 2020). Recently, this perspective was used to decide the appropriate time to release the COVID-19 lockdown, considering the costs and benefits in medical or social parameters (e.g., number of deaths, income) but also on mental health (Layard et al., 2020). In order to effectively manage future (and present) uncertain events such as the COVID-19, researchers and administrators of the mental health field would benefit from qualitative methodologies. For instance, online photovoice (OPV) is a form of participatory action research that enables individuals to express their strengths and concerns about their communities (Tanhan & Strack, 2020). In addition to all the specific recommendations to manage the mental health of the general population (Holmes et al., 2020) and healthcare workers (Greenberg, 2020; Greenberg et al., 2020), the present findings evidence the importance of including well-being in the assessment and improvement of mental health during the COVID-19 pandemic.

## Supplementary Information

Below is the link to the electronic supplementary material.

Supplementary file1 (DOCX 280 kb)

## Footnotes

1 Results did not vary after removing the studies with high risk of bias.

2 High levels of mental health using the Mental Health Continuum self-report.
