# Supplementary material for: Mental health among the general population and healthcare workers during the COVID-19 pandemic: A meta-analysis of well-being and psychological distress prevalence
Source: Curr Psychol. 2022 Mar 1:1–12. Online ahead of print. doi: 10.1007/s12144-022-02913-6 (PMC8887799; doi:10.1007/s12144-022-02913-6)
Supplement: Supplementary file 1 — Supplementary file1 (DOCX 280 kb) [file 12144_2022_2913_MOESM1_ESM.docx]

**Supplementary Materials**

**Table S1.**

*Search Strategy of all Databases*

| ***PUBMED:*** ("general population"[All Fields] OR "general public"[All Fields] OR ("communal"[All Fields] OR "communalism"[All Fields] OR "communalities"[All Fields] OR "communality"[All Fields] OR "communally"[All Fields] OR "commune"[All Fields] OR "communes"[All Fields] OR "community s"[All Fields] OR "communitys"[All Fields] OR "residence characteristics"[MeSH Terms] OR ("residence"[All Fields] AND "characteristics"[All Fields]) OR "residence characteristics"[All Fields] OR "communities"[All Fields] OR "community"[All Fields]) OR "adult*"[All Fields] OR "health workers"[All Fields] OR "health professionals"[All Fields]) AND ("severe acute respiratory syndrome coronavirus 2"[Supplementary Concept] OR "severe acute respiratory syndrome coronavirus 2"[All Fields] OR "ncov"[All Fields] OR "2019 ncov"[All Fields] OR "covid 19"[All Fields] OR "sars cov 2"[All Fields] OR (("coronavirus"[All Fields] OR "cov"[All Fields]) AND 2019/11/01:3000/12/31[Date - Publication]) OR "2019ncov"[All Fields] OR "hcov-19"[All Fields] OR ("severe acute respiratory syndrome coronavirus 2"[Supplementary Concept] OR "severe acute respiratory syndrome coronavirus 2"[All Fields] OR "sars cov 2"[All Fields]) OR ("sars virus"[MeSH Terms] OR ("sars"[All Fields] AND "virus"[All Fields]) OR "sars virus"[All Fields] OR ("sars"[All Fields] AND "cov"[All Fields]) OR "sars cov"[All Fields]) OR ("coronavirus"[MeSH Terms] OR "coronavirus"[All Fields] OR "coronaviruses"[All Fields]) OR "cov-19"[All Fields]) AND ("mental health"[All Fields] OR "psychological well-being"[All Fields] OR "social well-being"[All Fields] OR "emotional well-being"[All Fields] OR "subjective well-being"[All Fields] OR "wellbeing"[All Fields] OR ("health"[MeSH Terms] OR "health"[All Fields] OR "well"[All Fields] OR "well being"[All Fields]) OR "psychological distress"[All Fields] OR ("depressed"[All Fields] OR "depression"[MeSH Terms] OR "depression"[All Fields] OR "depressions"[All Fields] OR "depression s"[All Fields] OR "depressive disorder"[MeSH Terms] OR ("depressive"[All Fields] AND "disorder"[All Fields]) OR "depressive disorder"[All Fields] OR "depressivity"[All Fields] OR "depressive"[All Fields] OR "depressively"[All Fields] OR "depressiveness"[All Fields] OR "depressives"[All Fields]) OR ("anxiety"[MeSH Terms] OR "anxiety"[All Fields] OR "anxieties"[All Fields] OR "anxiety s"[All Fields]) OR ("stress"[All Fields] OR "stressed"[All Fields] OR "stresses"[All Fields] OR "stressful"[All Fields] OR "stressfulness"[All Fields] OR "stressing"[All Fields])). |
| --- |
| ***SCOPUS:*** (ALL ( "general population" OR "general public" OR community OR adult* OR "health workers" OR "health professionals" ) AND ALL ( covid-19 OR 2019ncov OR hcov-19 OR sars-cov-2 OR sars-cov OR coronavirus OR cov-19 ) AND ALL ( "Mental health" OR "psychological well-being" OR "social well-being" OR "emotional well-being" OR "subjective well-being" OR wellbeing OR well-being OR "psychological distress" OR depression OR anxiety OR stress ) AND ( LIMIT-TO ( SRCTYPE , "j" ) ) AND ( LIMIT-TO ( PUBSTAGE , "final" ) ) AND ( LIMIT-TO ( DOCTYPE , "ar" ) ) AND ( LIMIT-TO ( PUBYEAR , 2020 ) ) AND ( LIMIT-TO ( LANGUAGE , "English" ) OR LIMIT-TO ( LANGUAGE , "Spanish" ) ) AND ( LIMIT-TO ( EXACTKEYWORD , "Cross-sectional Study" ) ) AND ( LIMIT-TO ( EXACTKEYWORD , "Cross-sectional Studies" ) ) |
| ***PSYCINFO:*** (TX ( ("general population" OR "general public" OR community OR adult* OR "health workers" OR "health professionals") ) AND TX ( (covid-19 OR 2019ncov OR hcov-19 OR sars-cov-2 OR sars-cov OR coronavirus OR cov-19) ) AND TX ( (“mental health” OR “psychological well-being” OR “social well-being” OR “emotional well-being” OR “subjective well-being” OR wellbeing OR well-being OR “psychological distress” OR depression OR anxiety OR stress) ) |
| ***WEB OF SCIENCE:*** (TS=("general population" OR "general public" OR community OR adult* OR "health workers" OR "health professionals")) AND (TS=(covid-19 OR 2019ncov OR hcov-19 OR sars-cov-2 OR sars-cov OR coronavirus OR cov-19)) AND (TS=("mental health" OR "psychological well-being" OR "social well-being" OR "emotional well-being" OR "subjective well-being" OR wellbeing OR well-being OR "psychological distress" OR depression OR anxiety OR stress) ) |

**Table S2**

*Studies Included in the Systematic Review and Meta-Analysis (By Regions)*

| **Author, Country** | **Title** |  |
| --- | --- | --- |
| **Africa Region** | |  |
| Agberotimi et al., Nigeria | Interactions between socioeconomic status and mental health outcomes in the Nigerian context amid COVID-19 pandemic: A comparative study |  |
| **Region of the Americas** | |  |
| Arnetz, et al. US | Personal protective equipment and mental health symptoms among nurses during the COVID-19 pandemic |  |
| Barzilay, et al., US | Resilience, COVID-19-related stress, anxiety and depression during the pandemic in a large population enriched for healthcare providers |  |
| Bigalke, et al., US | Sex differences in self-report anxiety and sleep quality during COVID-19 stay-at-home orders |  |
| Campos, et al., Brazil | Early psychological impact of the COVID-19 pandemic in Brazil: A national survey |  |
| Czeisle, et al., US | Mental health, substance use, and suicidal ideation during the COVID-19 pandemic — United States, June 24–30, 2020 |  |
| Evanoff, et, al., US | Work-related and personal factors associated with mental well-being during the COVID-19 response: Survey of health care and other workers |  |
| Fitpatrick, et al., US | Living in the midst of fear: Depressive symptomatology among US adults during the COVID-19 pandemic |  |
| Gallagher, et al., US | The impact of Covid‐19 experiences and associated stress on anxiety, depression, and functional impairment in American adults |  |
| Hennein, et al., US | A hybrid inductive-abductive analysis of health workers' experiences and wellbeing during the COVID-19 pandemic in the United States |  |
| Liu, et al., US | Factors associated with depression, anxiety, and PTSD symptomatology during the COVID-19 pandemic: Clinical implications for U.S. young adult mental health |  |
| Passos, et al., Brazil | Impact on mental health due to covid-19 pandemic: Cross-sectional study in Portugal and Brazil |  |
| Pérez-Cano, et al., México | Anxiety, depression, and stress in response to the coronavirus disease-19 pandemic |  |
| Shechter, et al., US | Psychological distress, coping behaviors, and preferences for support among New York healthcare workers during the COVID-19 pandemic |  |
| Sherman, et al., US | Mental health outcomes associated with the COVID-19 pandemic: Prevalence and risk factors in a southern US state |  |
| Torales, et al., Paraguay | Self-perceived stress during the quarantine of COVID-19 pandemic in Paraguay: An exploratory survey |  |
| Twenge, et al., US | U.S. census bureau-assessed prevalence of anxiety and depressive symptoms in 2019 and during the 2020 COVID-19 pandemic |  |
| **South-east Asia Region** | |  |
| Al Bana, et al., Bangladesh | The impact of the COVID-19 pandemic on the mental health of the adult population in Bangladesh: a nationwide cross-sectional study |  |
| Chatterjee, et al., India | Attitude, practice, behavior, and mental health impact of COVID-19 on doctors |  |
| Chew, et al, India | A multinational, multicenter study on the psychological outcomes and associated physical symptoms amongst healthcare workers during COVID-19 outbreak |  |
| Groover, et al., India | Psychological impact of COVID-19 lockdown: An online survey from India |  |
| Khanal, et al., Nepal | Mental health impacts among health workers during COVID-19 in a low resource setting: A cross-sectional survey from Nepal |  |
| Margaretha, et al., Indonesia | Determinants psychological distress of Indonesian health care providers during COVID-19 pandemic |  |
| Pandey, et al., India | Psychological impact of mass quarantine on population during pandemics-The COVID-19 lock-down (COLD) study |  |
| Verma, et al., India | Depression, anxiety, and stress and socio-demographic correlates among general Indian public during COVID-19 |  |
| Shrestha, et al., Nepal | Mental wellbeing during the lockdown period following the COVID-19 pandemic in Nepal: A descriptive cross-sectional study |  |
| **European Region** |  |  |
| Alan, et al., Turkey | “I'm a hero, but…”: An evaluation of depression, anxiety, and stress levels of frontline healthcare professionals during COVID‐19 pandemic in Turkey |  |
| Amerio, et al., Italy | COVID-19 pandemic impact on mental health: A web-based cross-sectional on a sample of Italian general practitioners. |  |
| Antonijevic, et al., Serbia | Mental health of medical personnel during the COVID-19 pandemic |  |
| Azoulay, et al., Belgium | Symptoms of burnout in intensive care unit specialists facing the COVID-19 outbreak |  |
| Balsamo, et al., Italy | Italians on the age of COVID-19: The self-reported depressive symptoms through web-based survey |  |
| Bäuerle, et al., Germany | Mental health burden of the COVID-19 outbreak in Germany: Predictors of mental health impairment |  |
| Benke, et al., Germany | Lockdown, quarantine measures, and social distancing: Associations with depression, anxiety and distress at the beginning of the COVID-19 pandemic among adults from Germany |  |
| Blekas, et al., Greece | COVID-19: PTSD symptoms in Greek health care professionals |  |
| Bobes-Bascarán, et al., Spain | Early psychological correlates associated with COVID-19 in a Spanish older adult sample |  |
| Casagrande, et al., Italy | The enemy who sealed the world: effects quarantine due to the COVID-19 on sleep quality, anxiety, and psychological distress in the Italian population |  |
| Cunill, et al., Spain | The impact of COVID-19 on Spanish health professionals: A description of physical and psychological effects |  |
| Dawson, et al., UK | COVID-19: Psychological flexibility, coping, mental health, and wellbeing in the UK during the pandemic |  |
| Di Tella, et al., Italy | Mental health of healthcare workers during the COVID-19 pandemic in Italy |  |
| Elbay, et al., Turkey | Depression, anxiety, stress levels of physicians and associated factors in Covid-19 pandemics |  |
| Fiorillo, et al., Italy | Effects of the lockdown on the mental health of the general population during the COVID-19 pandemic in Italy: Results from the COMET collaborative network |  |
| Galindo-Vázquez, et al., Spain | Symptoms of anxiety, depression and self-care behaviors during the COVID-19 pandemic in the general population |  |
| Glowacz, et al., Belgium | Psychological distress during the COVID-19 lockdown: The young adults most at risk |  |
| Giusti, et al., Italy | The psychological impact of the COVID-19 outbreak on health professionals: A cross-sectional study |  |
| González-Sanguino, et al., Spain | Mental health consequences during the initial stage of the 2020 Coronavirus pandemic (COVID-19) in Spain |  |
| Günther-Bel, et al., Spain | A Mixed-method study of individual, couple, and parental functioning during the state-regulated COVID-19 lockdown in Spain |  |
| Gualano, et al., Italy | Effects of COVID-19 lockdown on mental health and sleep disturbances in Italy. |  |
| Hyland, et al., Ireland | Anxiety and depression in the Republic of Ireland during the COVID-19 pandemic |  |
| Horesh, et al., Israel | Risk factors for psychological distress during the COVID-19 pandemic in Israel: Loneliness, age, gender, and health status play an important role |  |
| Jia, et al., UK | Mental health in the UK during the COVID-19 pandemic: cross-sectional analyses from a community cohort study |  |
| Johnson, et al., Norway | PTSD symptoms among health workers and public service providers during the COVID-19 outbreak |  |
| Korkmaz, et al., Turkey | The anxiety levels, quality of sleep and life problem-solving skills in healthcare workers employed in COVID-19 services |  |
| Landi, et al., Italy | Health anxiety and mental health outcome during COVID-19 Lockdown in Italy: The mediating and moderating roles of psychological flexibility |  |
| Luceño-Moreno, et al., Spain | Symptoms of posttraumatic stress, anxiety, depression, levels of resilience and burnout in Spanish health personnel during the COVID-19 pandemic |  |
| Magnavita, et al., Italy | Symptoms in health care workers during the COVID-19 epidemic. A cross-sectional survey |  |
| Mazza, et al., Italy | A nationwide survey of psychological distress among Italian people during the covid-19 pandemic: Immediate psychological responses and associated factors |  |
| Milgrom, et al., Israel | Comparison of hospital worker anxiety in COVID-19 treating and non-treating hospitals in the same city during the COVID-19 pandemic |  |
| Mira, et al., Spain | Acute stress of the healthcare workforce during the COVID-19 pandemic evolution: A cross-sectional study in Spain |  |
| Munk, et al., Germany | COVID-19-Beyond virology: Potentials for maintaining mental health during lockdown |  |
| Ozamiz-Etxebarria, et al., Spain | Psychological symptoms during the two stages of lockdown in response to the COVID-19 outbreak: An investigation in a sample of citizens in Northern Spain |  |
| Ozamiz-Etxebarria, et al., Spain | Stress, anxiety, and depression levels in the initial stage of the COVID-19 outbreak in a population sample in the northern Spain. |  |
| Özdin, et al., Turkey | Levels and predictors of anxiety, depression and health anxiety during COVID-19 pandemic in Turkish society: The importance of gender |  |
| Papandreou, et al., Greece | Comparing eating behaviors, and symptoms of depression and anxiety between Spain and Greece during the COVID-19 outbreak: Cross-sectional analysis of two different confinement strategies |  |
| Papandreou, et al., Spain | Comparing eating behaviors, and symptoms of depression and anxiety between Spain and Greece during the COVID-19 outbreak: Cross-sectional analysis of two different confinement strategies |  |
| Parlapani, et al., Greece | Psychological and behavioral responses to the COVID-19 Pandemic in Greece |  |
| Passos, et al., Portugal | Impact on mental health due to covid-19 pandemic: Cross-sectional study in Portugal and Brazil |  |
| Pieh, et al., Austria | The effect of age, gender, income, work, and physical activity on mental health during coronavirus disease (COVID-19) lockdown in Austria |  |
| Robb, et al., UK | Associations of social isolation with anxiety and depression during the early COVID-19 Pandemic: A survey of older adults in London, UK |  |
| Rodríguez-Rey, et al., Spain | Psychological impact and associated factors during the initial stage of the coronavirus (COVID-19) pandemic among the general population in Spain |  |
| Rossi, et al., Italy | COVID-19 pandemic and lockdown measures impact on mental health among the general population in Italy |  |
| Rossi, et al., Italy | Mental health outcomes among frontline and second-line health care workers during the coronavirus disease 2019 (COVID-19) pandemic in Italy |  |
| Şahin, et al., Turkey | Prevalence of depression, anxiety, distress and insomnia and related factors in healthcare workers during COVID-19 pandemic in Turkey |  |
| Sandín, et al., Spain | Impacto psicológico de la pandemia de COVID-19: Efectos negativos y positivos |  |
| Shelvin, et al., UK | Anxiety, depression, traumatic stress and COVID-19-related anxiety in the UK general population during the COVID-19 pandemic |  |
| Solomou, et al., Cyprus | Prevalence and predictors of anxiety and depression symptoms during the COVID-19 pandemic and compliance with precautionary measures: Age and sex matter |  |
| Sonderskov, et al., Denmark | The depressive state of Denmark during the COVID-19 pandemic |  |
| Stylianou, et al., Cyprus | Mental health disorders during the COVID-19 outbreak in Cyprus |  |
| Ustun, et al., Turkey | Determining depression and related factors in a society affected by COVID-19 pandemic |  |
| Wánkowicz, et al., Poland | Assessment of mental health factors among health professionals depending on their contact with covid-19 patients |  |
| **Eastern Mediterranean** |  |  |
| AlAteeq, et al., Saudi Arabia | Mental health among healthcare providers during coronavirus disease (COVID-19) outbreak in Saudi Arabia |  |
| Alkhamees, et al., Saudi Arabia | The psychological impact of COVID-19 pandemic on the general population of Saudi Arabia |  |
| Alshekaili, et al., Oman | Factors associated with mental health outcomes across healthcare settings in Oman during COVID-19: Frontline versus non-frontline healthcare workers |  |
| Amin, et al., Pakistan | COVID-19 pandemic- knowledge, perception, anxiety and depression among frontline doctors of Pakistan |  |
| Arshad, et al., Pakistan | COVID-19 and anxiety amongst doctors: A Pakistani perspective |  |
| Burhamah, et al., Kuwait | The psychological burden of the COVID-19 pandemic and associated lockdown measures: Experience from 4000 participants |  |
| Hasan, et al., Pakistan | Anxiety among doctors during COVID-19 pandemic in secondary and tertiary care hospitals |  |
| Massad, et al., Jordan | The impact of the COVID-19 pandemic on mental health: Early quarantine-related anxiety and its correlates among Jordanians |  |
| Moghanibashi-Mansourieh, Iran | Assessing the anxiety level of Iranian general population during COVID-19 outbreak |  |
| Naser, et al., Jordan | Mental health status of the general population, healthcare professionals, and university students during 2019 coronavirus disease outbreak in Jordan: A cross-sectional study |  |
| Shatla, et al., Saudi Arabia | Public concerns and mental health changes related to the COVID-19 pandemic lockdown in Saudi Arabia |  |
| Temsah, et al., Saudi Arabia | The psychological impact of COVID-19 pandemic on healthcare workers in a MERS-CoV endemic country |  |
| Youssef, et al., Egypt | Mental health status of health-care professionals working in quarantine and non-quarantine Egyptian hospitals during the COVID-19 pandemic |  |
| Zandifar, et al., Iran | COVID-19 and medical staff's mental health in educational hospitals in Alborz Province, Iran |  |
| Zhang, et al., Iran | At the height of the storm: Healthcare staff's health conditions and job satisfaction and their associated predictors during the epidemic peak of COVID-19 |  |
| **Western Pacific** |  |  |
| An, et al., China | Prevalence of depression and its impact on quality of life among frontline nurses in emergency departments during the COVID-19 outbreak | |
| Awano, et al., Japan | Anxiety, depression, and resilience of healthcare workers in japan during the coronavirus disease 2019 outbreak | |
| Chen, et al., China | The psychological impact of COVID-19 outbreak on medical staff and the general public | |
| Cheng, et al., China | Anxiety in Chinese pediatric medical staff during the outbreak of Coronavirus Disease 2019: A cross-sectional study | |
| Chew, et al., Singapore | A multinational, multicenter study on the psychological outcomes and associated physical symptoms amongst healthcare workers during COVID-19 outbreak | |
| Every-Palmer, et al., New Zeland | Psychological distress, anxiety, family violence, suicidality, and wellbeing in New Zealand during the COVID-19 lockdown: A cross-sectional study | |
| Fauzi, et al., Malaysia | Doctors’ mental health in the midst of COVID-19 pandemic: The roles of work demands and recovery experiences | |
| Fisher, et al., Australia | Mental health of people in Australia in the first month of COVID-19 restrictions: a national survey | |
| Fu, et al., China | Psychological health, sleep quality, and coping styles to stress facing the COVID-19 in Wuhan, China | |
| Guo, et al., China | Mental health disorders and associated risk factors in quarantined adults during the COVID-19 outbreak in China: Cross-sectional study | |
| Gurvich, et al., Australia | Coping styles and mental health in response to societal changes during the COVID-19 pandemic | |
| Hou, et al., China | Gender differences of depression and anxiety among social media users during the COVID-19 outbreak in China: A cross-sectional study | |
| Hu, et al., China | Frontline nurses’ burnout, anxiety, depression, and fear statuses and their associated factors during the COVID-19 outbreak in Wuhan, China: A large-scale cross-sectional study | |
| Huang, et al., China | Chinese mental health burden during the COVID-19 pandemic | |
| Huang, et al., China | Generalized anxiety disorder, depressive symptoms and sleep quality during COVID-19 outbreak in China: A web-based cross-sectional survey | |
| Kuo, et al., Taiwan | Survey on perceived work stress and its influencing factors among hospital staff during the COVID-19 pandemic in Taiwan | |
| Lai, et al., China | Factors associated with mental health outcomes among health care workers exposed to Coronavirus Disease 2019 | |
| Lam, et al., China | Perceived risk and protection from infection and depressive symptoms among healthcare workers in Mainland China and Hong Kong during COVID-19 | |
| Lei, et al., China | Comparison of prevalence and associated factors of anxiety and depression among people affected by versus people unaffected by quarantine during the COVID-19 epidemic in Southwestern China | |
| Li, et al., China | Anxiety and related factors in frontline clinical nurses fighting COVID-19 in Wuhan | |
| Li, et al., China | Factors associated with mental health results among workers with income losses exposed to COVID‐19 in China | |
| Li, et al., China | The psychological health status of healthcare workers during the COVID-19 pandemic outbreak: A cross-sectional survey study in Guangdong, China | |
| Liang, et al., China | Mental health in frontline medical workers during the 2019 novel coronavirus disease epidemic in China: A comparison with the general population | |
| Liu, et al., China | The prevalence and influencing factors in anxiety in medical workers fighting COVID-19 in China: A cross-sectional survey | |
| Liu, et al., China | Mental health status of pediatric medical workers in China during the COVID-19 outbreak | |
| Lu, et al., China | Psychological status of medical workforce during the COVID-19 pandemic: A cross-sectional study | |
| Lu, et al., China | The psychological states of people after Wuhan eased the lockdown | |
| Ma, et al., China | Psychological stress among health care professionals during the 2019 novel coronavirus disease outbreak: Cases from online consulting customers | |
| Na, et al., China | Mental health, risk factors, and social media use during the COVID-19 epidemic and cordon sanitaire among the Community and Health Professionals in Wuhan, China: Cross-sectional survey | |
| Newby, et al., Australia | acute mental health responses during the COVID-19 pandemic in Australia | |
| Park, et al., Korea | Psychological distress among infectious disease physicians during the response to the COVID-19 outbreak in the Republic of Korea | |
| Perveen, et al., Malaysia | Prevalence of anxiety, stress, depression among Malaysian adults during COVID-19 pandemic movement control order | |
| Qian, et al., China | Anxiety levels, precautionary behaviors and public perceptions during the early phase of the COVID-19 outbreak in China: A population-based cross-sectional survey | |
| Que, et al., China | Psychological impact of the covid-19 pandemic on healthcare workers: A cross-sectional study in China | |
| Ran, et al., China | Psychological resilience, depression, anxiety, and somatization symptoms in response to COVID-19: A study of the general population in China at the peak of its epidemic | |
| Ren, et al., China | The psychological burden experienced by Chinese citizens during the COVID-19 outbreak: prevalence and determinants | |
| Ren, et al., China | Public mental health under the long-term influence of COVID-19 in China: Geographical and temporal distribution | |
| Shi, et al., China | Prevalence of and risk factors associated with mental health symptoms among the general population in China during the Coronavirus disease 2019 pandemic | |
| Si, et al., China | Psychological impact of COVID-19 on medical care workers in China | |
| Song, et al., China | Mental health status of medical staff in emergency departments during the Coronavirus disease 2019 epidemic in China | |
| Song, et al., China | Mental health and work attitudes among people resuming work during the COVID-19 pandemic: A cross-sectional study in China | |
| Su, et al., China | Proximity to people with COVID-19 and anxiety among community residents during the epidemic in Guangzhou, China | |
| Stanton, et al., Australia | Depression, anxiety and stress during COVID-19: Associations with changes in physical activity, sleep, tobacco and alcohol use in Australian adults | |
| Tang, et al., China | COVID-19 related depression and anxiety among quarantined respondents | |
| Tee, et al., Phillipines | Psychological impact of COVID-19 pandemic in the Philippines | |
| Teng, et al., China | Psychological status and fatigue of frontline staff two months after the COVID-19 pandemic outbreak in China: A cross-sectional study | |
| Tian, et al., China | Psychological symptoms of ordinary Chinese citizens based on SCL-90 during the level I emergency response to COVID-19 | |
| Tu, et al., China | Sleep quality and mood symptoms in conscripted frontline nurse in Wuhan, China during COVID-19 outbreak: A cross-sectional study | |
| Wang, et al., China | Immediate psychological responses and associated factors during the initial stage of the 2019 Coronavirus disease (COVID-19) epidemic among the general population in China | |
| Wang, et al., China | Psychological distress and sleep problems when people are under interpersonal isolation during an epidemic: A nationwide multicenter cross-sectional study | |
| Wang, et al., China | Sleep disturbance and psychological profiles of medical staff and non-medical staff during the early outbreak of COVID-19 in Hubei Province, China | |
| Wang, et al., China | Acute psychological effects of Coronavirus disease 2019 outbreak among healthcare workers in China: A cross-sectional study | |
| Wang, et al., China | Psychological impact of coronavirus disease (2019) (COVID-19) epidemic on medical staff in different posts in China: A multicenter study | |
| Wong, et al., China | Anxiety symptoms and preventive measures during the COVID-19 outbreak in Taiwan | |
| Xiamonig, et al., China | The psychological status of 8817 hospital workers during COVID-19 Epidemic: A cross-sectional study in Chongqing | |
| Xiao, et al., China | Psychological impact of healthcare workers in China during COVID-19 pneumonia epidemic: A multi-center cross-sectional survey investigation | |
| Xing, et al., China | Study of the mental health status of medical personnel dealing with new coronavirus pneumonia | |
| Xiong, et al., China | The psychological status and self-efficacy of nurses during COVID-19 outbreak: A cross-sectional survey | |
| Zhan, et al., China | Prevalence and influencing factors on fatigue of first-line nurses combating with COVID-19 in China: A descriptive cross-sectional study | |
| Zhang, et al., China | Status and influential factors of anxiety depression and insomnia symptoms in the work resumption period of COVID-19 epidemic: A multicenter cross-sectional study | |
| Zhang, et al., China | Prevalence of anxiety and depression symptoms, and association with epidemic-related factors during the epidemic period of COVID-19 among 123,768 workers in China: A large cross-sectional study | |
| Zhou, et al., China | The prevalence and risk factors of psychological disturbances of frontline medical staff in china under the COVID-19 epidemic: Workload should be concerned | |
| Zhu, et al., China | Prevalence and influencing factors of anxiety and depression symptoms in the first-line medical staff fighting against COVID-19 in Gansu | |
| Zhu, et al., China | The mediation effects of coping style on the relationship between social support and anxiety in Chinese medical staff during COVID-19 | |
| Zhu, et al., China | COVID-19 in Wuhan: Sociodemographic characteristics and hospital support measures associated with the immediate psychological impact on healthcare workers | |

**Table S3.**

*Main Characteristics of the Included Studies (k = 158; N = 880,352)*

| First author (year) | Country | Group | Sample size (N) | Females (%) | M_age_ (SD) | Outcomes | Assessment tool | Prevalence (%) |
| --- | --- | --- | --- | --- | --- | --- | --- | --- |
| Agberotimi (2020) | Nigeria | GP  HCWs | GP 502  HCWs 382 | GP 44.8%  HCWs 44.2% | 28.75 (8.17) | Depression, Anxiety | PHQ-9, GAD-7 | *GP:*  Depression 23.5%  Anxiety 49.6%  *HCWs:*  Depression 35.1%  Anxiety 58.4% |
| Al Banna (2020) | Bangladesh | GP | 1427 | 28.5% | ≥18-65 | Depression, Anxiety | DASS-21 | Anxiety 33.7%  Depression 57.9% |
| Alan (2020) | Turkey | HCWs | 416 | 79.08% | 33.6 (8.7) | Depression, Anxiety, Stress | DASS | Depression 52.7%  Anxiety 54.3%  Stress 44.2% |
| AlAteeq (2020) | Saudi Arabia | HCWs | 502 | 31.9% | ≥18 | Depression, Anxiety | PHQ-9,  GAD-7 | Depression 30.3%  Anxiety 26.3% |
| Alkhamees (2020) | Saudi Arabia | GP | 1160 | 63.9% | ≥18 | Depression, Anxiety | DASS-21 | Depression 28.3%  Anxiety 24% |
| Alshekaili (2020) | Oman | HCWs | 1139 | 80% | 36.3 (6.5) | Depression, Anxiety, Stress | DASS-21 | Depression 32.3%  Anxiety 34.1%  Stress 23.8% |
| Alzueta (2020) | 59 countries | GP | 6882 | 78.8% | 42.30 (13.95) | Depression, Anxiety | DASS-21, GAD-7 | Depression 25.4%  Anxiety 19.5% |
| Amin (2020) | Pakistan | HCWs | 250 | 63.6% | ≥20 | Well-being | PGWBI | Well-being 28.6% |
| An (2020) | China | HCWs | 1103 | 90.8% | 32.20 (7.61) | Depression | PHQ | Depression 43.6% |
| Amerio (2020) | Italy | GP | 131 | 48.1% | 52.31 (12.24) | Depression | PHQ-9 | Depression 22.9% |
| Antonijevic (2020) | Serbia | GP  HCWs | 1678 | 78.4% | 40.38 (10.32) | Depression, Anxiety, Stress | BDI‐IA, GAD-7, PSS | *GP:*  Depression 19.1%  Anxiety 56.7%  Stress 65%  *HCWs:*  Depression 18.2%  Anxiety 71.6%  Stress 74.9% |
| Arnetz (2020) | USA | HCWs | 695 | 93.6% | ≥18 | Depression, Anxiety | PHQ-9, GAD-7 | Depression 59.5%  Anxiety 54.9% |
| Arsha (2020) | Pakistan | HCWs | 431 | 44.78% | ≥18 | Anxiety | GAD-7 | Anxiety 33.6% |
| Awano (2020) | Japan | HCWs | 848 | 74.88% | 37 (NR) | Depression, Anxiety | GAD-7, CES-D | Depression 27.9%  Anxiety 10% |
| Azoulay (2020) | Belgium | HCWs | 848 | 34% | ≤35 | Depression, Anxiety | HADS | Depression 30.2%  Anxiety 46.5% |
| Balsamo (2020) | Italy | GP | 3672 | 65.1% | 33.27 (14.29) | Depression, Anxiety | TDI, STICSA | Depression 37.1%  Anxiety 19.8% |
| Barzilay (2020) | USA | GP  HCWs | 3042 | 64.56% | 18-79 | Depression, Anxiety | PHQ-2, GAD-7 | Depression 16.1%  Anxiety 22.2% |
| Bäuerle (2020) | Germany | GP | 15037 | 70.7% | ≥18 | Depression, Anxiety | PHQ-2, GAD-2 | Depression 14.3%  Anxiety 19.7% |
| Benke (2020) | Germany | GP | 4335 | 75.8% | 40.50 (12.45) | Depression, Anxiety | PHQ-9, GAD-7 | Depression 31.1%  Anxiety 21.2% |
| Bigalke (2020) | USA | GP | 103 | 59% | 18-68 | Anxiety | STAI | Anxiety 68% |
| Blekas (2020) | Greece | HCWs | 270 | 73.7% | 37.61 (11.93) | Depression | PHQ-9 | Depression: 11.1% |
| Bobes-Bascarán (2020) | Spain | GP | 2194 | 54.6% | 65.62 (5.05) | Depression, Anxiety, Stress | DASS-21 | Depression 7.4%  Anxiety 1.7%  Stress 7% |
| Bressington (2020) | China | GP | 11072 | 80.7% | ≥18 | Depression | PHQ-9 | Depression 46.5% |
| Burhamah (2020) | Kuwait | GP | 4132 | 69.3% | ≤20-71 | Depression, Anxiety | PHQ-9, GAD-7 | Depression 30.1%  Anxiety 26.3% |
| Campos (2020) | Brazil | GP | 12196 | 69.8% | 35.2 (13) | Depression, Anxiety, Stress | DASS-21, IES-R | Depression 46.8%  Anxiety 35.7%  Stress 35.3% |
| Casagrande (2020) | Italy | GP | 2291 | 74.6% | ≥18 | Anxiety | GAD-7 | Anxiety 32.1% |
| Chatterjee (2020) | India | HCWs | 152 | 21.7% | 42.05 (12.19) | Depression, Anxiety, Stress | DASS-21 | Depression 24.3%  Anxiety 41.6%  Stress 19.7% |
| Chen (2020) | China | GP  HCWs | GP 1071  HCWs 422 | 44.61% | ≥18 | Depression, Stress | SDS  PSS-21 | *GP:*  Depression 2.9%  Stress 86.7%  *HCWs*  Depression 3.8%  Stress 93.4% |
| Cheng (2020) | China | HCWs | 534 | 82.4% | ≥18 | Anxiety | SAS | Anxiety 3.3% |
| Chew (2020) | Singapore and India | HCWs | 906 | 64.3% | 29 (NR) | Depression, Anxiety, Stress | DASS-21 | Depression 10.6%  Anxiety 15.7%  Stress 5.2% |
| Cunill (2020) | Spain | HCWs | 1452 | 82.9% | ≤30 | Depression, Anxiety | PHQ-9, GAD-7 | Depression 86.1%  Anxiety 88.4% |
| Czeisler (2020) | USA | GP | 5470 | 50.9% | 18- ≥65 | Anxiety | PHQ | Anxiety 31% |
| Dawson (2020) | UK | GP | 555 | 72% | 39.2 (13.2) | Depression, Anxiety | PHQ-9, GAD-7 | Depression 37%  Anxiety 27% |
| Di Tella (2020) | Italy | HCWs | 145 | 72.4% | 42.9 (11.2) | Depression, Anxiety | STAI Y1, BDI-II | Depression 31%  Anxiety 71% |
| Elbay (2020) | Turkey | HCWs | 442 | 56.8% | 36.05 (8.69) | Depression, Anxiety, Stress | DASS-21 | Depression 47.1%  Anxiety 35.2%  Stress 31% |
| Evanoff (2020) | USA | GP | 5550 | 78.0% | ≥40 | Depression, Anxiety | DASS-21 | Depression 15.9%  Anxiety 13% |
| Every-Palmer (2020) | New Zealand | GP | 2010 | 52.9% | ≥18 | Anxiety, Well-being | GAD-7, WHO-5 | Anxiety 16%  Well-being 61% |
| Fauzi (2020) | Malaysia | HCWs | 1050 | 71.5% | 33.08 (6.96) | Depression, Anxiety, Stress | DASS-21 | Depression 17.2%  Anxiety 21.8%  Stress 14% |
| Fiorillo (2020) | Italy | GP | 20720 | 71% | 40.4 (14.3) | Depression, Anxiety, Stress | DASS-21 | Depression 48.9%  Anxiety 34.3%  Stress 41.6% |
| Fisher (2020) | Australia | GP | 13829 | 75.5% | ≥18 | Depression, Anxiety | PHQ-9, GAD-7 | Depression 27.6%  Anxiety 21% |
| Fitzpatrick (2020) | USA | GP | 10368 | 51.02% | 47 (18) | Depression | CES-D | Depression 33% |
| Fu (2020) | China | GP | 1242 | 69.73% | ≥18 | Depression, Anxiety | PHQ-9, GAD-7 | Depression 29.3%  Anxiety 27.5% |
| Galindo-Vázquez (2020) | Spain | GP | 1508 | 86.4% | ≥18 | Depression, Anxiety | PHQ-9, GAD-7 | Depression 27.5%  Anxiety 20.8% |
| Gallagher (2020) | USA | GP | 565 | 33.1% | 38.26 (12.13) | Depression, Anxiety | ODSIS, OASIS | Depression 31.3%  Anxiety 38.4% |
| Giusti (2020) | Italy | HCWs | 330 | 62.6% | 44.6 (13.5) | Depression, Anxiety | STAI-S, DASS-21 | Depression 26.8%  Anxiety 31.3% |
| Glowacz (2020) | Belgium | GP | 2871 | 79.03% | ≥18 | Depression, Anxiety | HADS | Depression 84.3%  Anxiety 35.7% |
| González-Sanguino (2020) | Spain | GP | 3480 | 75% | 37.92 (NR) | Depression, Anxiety | PHQ-2, GAD-2 | Depression 18.7%  Anxiety 21.6% |
| Grover (2020) | India | GP  HCWs | GP 891  HCWs 794 | 36.3% | 41.26 (13.67) | Depression, Anxiety, Stress | PHQ-9, GAD-7, PSS  WEMWBS | *GP:*  Depression 11.8%  Anxiety 15.7%  Stress 74.7%  Well-being 30.8%  HCWs:  Depression 9%  Anxiety 13%  Stress 73.3%  Well-being 25.6% |
| Gualano (2020) | Italy | GP | 1515 | 65.6% | 42 (NR) | Depression, Anxiety | PHQ-2, GAD-2 | Depression 24.7%  Anxiety 23.2% |
| Günther-Bel (2020) | Spain | GP | 407 | 77% | 42.7 (12.7) | Depression, Anxiety | STAI, BDI | Depression 13.4%  Anxiety 71.9% |
| Gurvich (2020) | Australia | GP | 1495 | 81.6% (1226) | M= 42.5 | Depression, Anxiety, Stress | DASS-21 | Depression: 54.7%  Anxiety: 29.7%  Stress: 37.2% |
| Guo (2020) | China | GP | 2331 | 56.1% | 34.4 (11.1) | Depression, Anxiety | HADS | Depression 21.3%  Anxiety 25.4% |
| Hasan (2020) | Pakistan | HCWs | 151 | 56.3% | 29 (7.28) | Anxiety | GAD-7 | Anxiety 17.9% |
| Hennein (2020) | USA | HCWs | 1132 | 71.4% | ≥18 | Depression, Anxiety | PHQ-9, GAD-7 | Depression 14%  Anxiety 15.8% |
| Horesh (2020) | Israel | GP | 204 | 71.1% | 21-84 | Anxiety, Stress | PSS, BAI | Stress 61.6%  Anxiety 89.1% |
| Hou (2020) | China | GP | 3063 | 56.7% | 37.73 (13.38) | Depression, Anxiety | PHQ-2, GAD-2 | Depression 14.1%  Anxiety 13.3% |
| Hu (2020) | China | HCWs | 2014 | 87.1% | 30.99 (6.17) | Depression, Anxiety | SDS, SAS | Depression 10.7%  Anxiety 14.3% |
| Huang (2020a) | China | GP | 7236 | 54.6% | 35.3 (5.6) | Depression, Anxiety | GAD-7 | Depression 20.1%  Anxiety 35.1% |
| Huang (2020b) | China | GP | 1172 | 69.3% | 28.39 (10.49) | Anxiety | GAD-7 | Anxiety 33% |
| Hyland (2020) | Republic of Ireland | GP | 1041 | 51.5% | 44 (15.76) | Depression, Anxiety | GAD-7, PHQ-9 | Depression 22.8%  Anxiety 20% |
| Jia (2020) | UK | GP | 3097 | 84.5% | 44 (NR) | Depression, Anxiety | PHQ-9, GAD-7 | Depression 31.6%  Anxiety 26% |
| Johnson (2020) | Norway | HCWs | 298 | 84.7% | ≥18 | Depression, Anxiety | PHQ-9, GAD-7 | Depression 26.2%  Anxiety 23.8% |
| Khanal (2020) | Nepal | HCWs | 475 | 52.6% | 28.2 (5.80) | Depression, Anxiety | HADS | Depression 13.5%  Anxiety 18.3% |
| Korkmaz (2020) | Turkey | HCWs | 140 | 44% | 18-65 | Anxiety | BAI | Anxiety 33% |
| Kuo (2020) | Taiwan | HCWs | 752 | 88.6% | 38.7 (9.3) | Stress | PEHWSRPSHID | Stress 17.15% |
| Lai (2020) | China | HCWs | 1257 | 76.7% | ≥18 | Depression, Anxiety | PHQ-9, GAD-7 | Depression 14.8%  Anxiety 12.3% |
| Lam (2020) | China | HCWs | 932 | 73.43% | ≥18 | Depression | PHQ-9 | Depression 78.4% |
| Landi (2020) | Italy | GP | 944 | 73.5% | 38.86 (13.2) | Depression, Anxiety | PHQ-9, GAD-7 | Depression 23.4%  Anxiety 18.1% |
| Lei (2020) | China | GP | 1593 | 61.3% | 32.3 (9.8) | Depression, Anxiety | SAS, SDS | Depression 6.3%  Anxiety 2.5% |
| Li (2020) | China | HCWs | 908 | 57.55% | 33.8 (6.93) | Depression, Anxiety | SAS, SDS | Depression 32.9%  Anxiety 24.3% |
| Li (2020) | China | HCWs | 176 | 77.3% | ≥18 | Anxiety | HAMA | Anxiety 50% |
| Li (2020) | China | GP | 398 | 49.5% | ≥18 | Depression, Anxiety | PHQ-9, GAD-7 | Depression 45.5%  Anxiety 49.5% |
| Liang (2020) | China | GP  HCWs | GP 1104  HCWs 899 | GP 69.47%  HCWs 81.31% | ≥18 | Depression, Anxiety | PHQ-9, GAD-7 | *GP:*  Depression 20.9%  Anxiety 12.9%  *HCWs:*  Depression 26.8%  Anxiety 16.7% |
| Liu (2020) | USA | GP | 898 | 81.3% | 24.5 (NR) | Depression, Anxiety | PHQ-9, GAD-7 | Depression 43.3%  Anxiety 45.4% |
| Liu (2020) | China | HCWs | 512 | 84.57% | ≥18 | Anxiety | SAS | Anxiety 25% |
| Liu (2020) | China | HCWs | 2031 | 85.52% | ≥18 | Depression, Anxiety, Stress | DASS | Depression 14.8%  Anxiety 18.3%  Stress 10% |
| Lu (2020) | China | GP  HCWs | GP 1035  HCWs 387 | 83.4% | ≥18 | Depression, Anxiety | PHQ-9, GAD-7 | *GP*  Depression 21.2%  Anxiety 16.7%  *HCWs*  Depression 16.1%  Anxiety 22.3% |
| Lu (2020) | China | HCWs | 2042 | 77.9% | ≤30 | Depression, Anxiety | HAMA, HAMD | Depression 12.1%  Anxiety 25.5% |
| Luceño-Moreno (2020) | Spain | HCWs | 1228 | 86.4% | 19-68 | Depression, Anxiety | HADS | Depression 51.3%  Anxiety 79.3% |
| Ma (2020) | China | HCWs | 34 | 24 | 18-40 | Depression, Anxiety, Stress | PHQ-9, GAD-7 | Depression 35%  Anxiety 24% |
| Magnavita (2020) | Italy | HCWs | 595 | 70% | ≥18 | Depression, Anxiety | GADS | Depression 20.3%  Anxiety 16.6% |
| Margaretha (2020) | Indonesia | HCWs | 682 | 71.8% | ≥18 | Depression, Anxiety, Stress | DASS-21 | Depression 7.5%  Anxiety: 17.7%  Stress 15.5% |
| Massad (2020) | Jordan | GP | 5274 | 55.3% | ≥18 | Anxiety | BAI | Anxiety 16.9% |
| Mazza (2020) | Italy | GP | 2766 | 71.6% | 32.94 (13.2) | Depression, Anxiety, Stress | DASS-21 | Depression 32.8%  Anxiety 18.7%  Stress 27.2% |
| Milgrom (2020) | Israel | HCWs | 1570 | - | ≥18 | Anxiety | STAI-S | Anxiety 33.5% |
| Mira (2020) | Spain | HCWs | 685 | - | ≥18 | Stress | EASE | Stress 28.4% |
| Moghanibashi-Mansourieh (2020) | Iran | GP | 10754 | 65.8% | ≥18 | Anxiety | DASS-21 | Anxiety 40.4% |
| Munk (2020) | Germany | GP | 949 | 79.5% | 28.9 (10.8) | Depression, Anxiety | GAD, DEP | Depression 35.3%  Anxiety 12% |
| Naser (2020) | Jordan | GP  HCWs | 2961 | 59% | ≥18 | Depression, Anxiety | PHQ-9, GAD-7 | *GP:*  Anxiety 8.3%  Depression 15.8%  *HCWs:*  Depression 21.2%  Anxiety 11.3% |
| Newby (2020) | Australia | GP | 5071 | 85.8% | ≥18 | Depression, Anxiety, Stress | DASS-21 | Depression 46.3%  Anxiety 40.8%  Stress 38.7% |
| Ni (2020) | China | GP  HCWs | GP 1577  HCWs 214 | GP 60.8%  HCWs 68.8% | ≥18 | Depression, Anxiety | PHQ-2, GAD-2 | *GP:*  Depression 19.21%  Anxiety 23.84%  *HCWs:*  Depression 22%  Anxiety 19.2% |
| Ozamiz-Etxebarria (2020) | Spain | GP | 737 | 74.76% | ≥18 | Depression, Anxiety, Stress | DASS | Depression 8.9%  Anxiety 18.5%  Stress 13.6% |
| Ozamiz-Etxebarria (2020) | Spain | GP | 1993 | 79.5% | 18-82 | Depression, Anxiety, Stress | DASS-21 | Depression 27.5%  Anxiety 26.9%  Stress 26.5% |
| Özdin (2020) | Turkey | GP | 343 | 49.2% | 37.16 (10.31) | Depression, Anxiety | HADS | Depression 23.6%  Anxiety 45.1% |
| Pandey (2020) | India | GP | 1395 | 58.1% | 18-64 | Depression, Anxiety, Stress | DASS-21 | Depression 30.5%  Anxiety 22.4%  Stress 10.8% |
| Papandreou (2020) | Spain and Greece | GP | Spain 1002 Greece 839 | - | Spain  46.1 (13.3)  Greece  42.4 (11.7) | Depression, Anxiety | PHQ-9, GAD-7 | *Spain:*  Depression 13.6%  Anxiety 12.3%  *Greece:*  Depression 18.8%  Anxiety 13.2% |
| Park (2020) | Republic of Korea | HCWs | 265 | 58.3% | 41 (37–48) | Depression, Anxiety, Stress | DASS | Depression 17.4%  Anxiety 20%  Stress 4.3% |
| Parlapani (2020) | Greece | GP | 3029 | 71.9% | ≥18 | Depression, Anxiety | PHQ-9, GAD-7 | Depression 22.8%  Anxiety 77.4% |
| Passos (2020) | Portugal and Brazil | GP | 550 | 79.5% | 38 (NR) | Depression, Anxiety | GAD-7, PHQ-2 | *Portugal*:  Depression 22.6%  Anxiety 28.1%  *Brazil*:  Depression 26.6%  Anxiety 74.7% |
| Pérez-Cano (2020) | Mexico | GP | 613 | 76% | 26.77 (10.30) | Depression, Anxiety, Stress | DASS-21, STAI | Depression 41.3%  Anxiety 42%  Stress 26.8% |
| Perveen (2020) | Malaysia | GP | 716 | 73.04% | 18-69 | Depression, Anxiety, Stress | DASS | Depression 19.9%  Anxiety 40.1%  Stress 19.2% |
| Pieh (2020) | Austria | GP | 1005 | 52.7% | ≥18 | Depression, Anxiety | PHQ-9, GAD-7 | Depression: 21%  Anxiety: 19% |
| Qian (2020) | China | GP | 1011 | 50.65% | ≥18 | Anxiety | GAD-7 | Anxiety 26.6% |
| Que (2020) | China | HCWs | 2285 | 63.49% | 31.06 (6.99) | Depression, Anxiety | PHQ-9, GAD-7 | Depression 44.4%  Anxiety 46% |
| Ran (2020) | China | GP | 1770 | 66.9% | 28.70 (10.64) | Depression, Anxiety, Stress | PHQ-9, PHQ-15, GAD-7 | Depression 47.1%  Anxiety 31.9%  Stress 45.9% |
| Ren (2020) | China | GP | 1172 | 69.3% | 22.0 (21.0–37.0) | Depression, Anxiety, Stress | PHQ-9, GAD-7, PSS-10 | Depression 18.8%  Anxiety 13.3%  Stress 67.9% |
| Ren (2020) | China | GP | 6130 | 66.9% | ≥18 | Depression, Anxiety | PHQ-9, GAD-7 | Depression 12%  Anxiety 7.1% |
| Robb (2020) | UK | GP | 7127 | 54.1% | 70.7 (7.4) | Depression, Anxiety | HADS | Depression 2.5%  Anxiety 5.5% |
| Rodríguez-Rey (2020) | Spain | GP | 3055 | 75.1% | ≥18 | Depression, Anxiety, Stress | DASS-21, IES-R | Depression 41%  Anxiety 25%  Stress 41% |
| Rossi (2020) | Italy | HCWs | 1379 | 77.2% | 39 (16) | Depression, Anxiety, Stress | PHQ-9, GAD-7, PSS-10 | Depression 24.7%  Anxiety 19.8%  Stress 21.9% |
| Rossi (2020) | Italy | GP | 18147 | 79.5% | 38 (NR) | Depression, Anxiety, Stress | PHQ-9, GAD-7, PSS | Depression 17.3%  Anxiety 20.8%  Stress 21.9% |
| Şahin (2020) | Turkey | HCWs | 939 | 66% | ≥18 | Depression, Anxiety, Stress | PHQ-9, GAD-7, IESR | Depression 77.6%  Anxiety 60.2%  Stress 76.4% |
| Sandín (2020) | Spain | GP | 1161 | 77.3% | 33.4 (10.7) | Depression, Anxiety, Stress | ED | Depression 9.9%  Anxiety 9.5%  Stress 12.5% |
| Shatla (2020) | Saudi Arabia | GP | 1921 | 84.7% | ≥18 | Depression, Anxiety | HADS | Depression 37.3%  Anxiety 26.4% |
| Shechter (2020) | USA | HCWs | 657 | 70.9% | 18-≥75 | Depression, Anxiety, Stress | PHQ-2, GAD-2, PC-PTSD | Depression 48%  Anxiety 33%  Stress 57% |
| Sherman (2020) | USA | GP | 591 | 77.50% | 51.19 (14.81) | Depression, Anxiety | PHQ-9, GAD-7 | Depression 21%  Anxiety 16.6% |
| Shevlin (2020) | UK | GP | 2025 | 51.7% | 45 (15.9) | Depression, Anxiety | GAD-7, PHQ-9 | Depression 22.1%  Anxiety 21.6% |
| Shi (2020) | China | GP | 56932 | 52.1% | ≥18 | Depression, Anxiety, Stress | PHQ-9, GAD-7, ASDS | Depression 27.9%  Anxiety 31.6%  Stress 24.4% |
| Shrestha (2020) | Nepal | HCWs | 254 | 48.7% | 25.93 (6.88) | Well-being | WHO-5 | Well-being 61% |
| Si (2020) | China | HCWs | 863 | 70.7% | ≥18 | Depression, Anxiety, Stress | DASS, IES-6 | Depression 13.6%  Anxiety 13.9%  Stress 8.6% |
| Solomou (2020) | Cyprus | GP | 1642 | 71.6% | ≥18 | Depression, Anxiety | PHQ-9, GAD-7 | Depression 57.2%  Anxiety 64.1% |
| Sønderskov (2020) | Denmark | GP | 2458 | 51% | 49.1 (NR) | Well-being | WHO-5 | Well-being 26.4% |
| Song (2020) | China | GP | 709 | 74.2% | 35.35 (6.61) | Depression, Anxiety | CES-D, GAD-7 | Depression 13.5%  Anxiety 14.1% |
| Song (2020) | China | HCWs | 14825 | 64.3% | 34 (8.2) | Depression | CES-D | Depression 25.2% |
| Stanton (2020) | Australia | GP | 1491 | 67.4% | 50.5 (14.9) | Depression, Anxiety, Stress | DASS-21 | Depression 26.5%  Anxiety 13.5%  Stress 18.1% |
| Stylianou (2020) | Cyprus | GP | 193 | 48.15% | ≥18 | Depression, Anxiety | PHQ-9, GAD-7 | Depression 7.9%  Anxiety 15.8% |
| Su (2020) | China | GP | 403 | 68.5% | 42 (11.2) | Anxiety | GAD-7 | Anxiety 37.7% |
| Tang (2020) | China | GP | 1160 | 60% | ≥18 | Depression, Anxiety | CES-D-20, GAD-7 | Depression 26.5%  Anxiety:70.8% |
| Tee (2020) | Philippines | GP | 1879 | 69% | ≥21 | Depression, Anxiety, Stress | DASS-21, IES-R | Depression 16.9%  Anxiety 28.8%  Stress 13.4% |
| Temsah (2020) | Saudi Arabia | HCWs | 811 | 75.1% | ≤30 | Anxiety | GAD-7 | Anxiety 31.8% |
| Teng (2020) | China | HCWs | 2614 | 55.6% | ≥18 | Depression, Anxiety | PHQ-9, SAS | Depression 59%  Anxiety 57.5% |
| Tian (2020) | China | GP | 1132 | 84.7% | 18-76 | Depression, Anxiety | SCL-90 | Depression 43.1%  Anxiety 39.9% |
| Torales (2020) | Paraguay | GP | 2206 | 25.9% | 18-75 | Stress | EEP-10 | Stress 67.5% |
| Tu (2020) | China | HCWs | 100 | 100% | 34.44 (5.85) | Depression, Anxiety | PHQ-9, GAD-7 | Depression 46%  Anxiety 40% |
| Twenge (2020) | USA | GP | 319458 |  | ≥18 | Depression, Anxiety | PHQ-2, GAD-2 | Depression 24.2%  Anxiety 29.6% |
| Ustun (2021) | Turkey | GP | 1115 | 71.7% | 27.98 (8.79) | Depression | BDI | Depression 27.3% |
| Verma (2020) | India | GP | 354 | 48.3% | ≥18 | Depression, Anxiety, Stress | DASS-21 | Depression 25.1%  Anxiety 28%  Stress 11.6% |
| Wang (2020) | China | GP | 1210 | 67.3% | ≥18 | Depression, Anxiety, Stress | DASS-21 | Depression 16.5%  Anxiety 28.8%  Stress 8.1% |
| Wang (2020) | China | HCWs | 274 | 77.4% | 22-64 | Depression, Anxiety | PHQ-9, GAD-7 | Depression 16.1%  Anxiety 13.9% |
| Wang (2020) | China | GP | 19372 | 52% | 18-87 | Depression, Anxiety | PHQ-9, GAD-7 | Depression 13.3%  Anxiety 11% |
| Wang (2020) | China | HCWs | 2737 | 64.5% | 18-65 | Depression, Anxiety | HADS-A, HADS-D | Depression 35%  Anxiety 22.6% |
| Wang (2020) | China | HCWs | 1897 | 82.5% | ≥18 | Depression, Anxiety | PHQ-9, GAD-7 | Depression 15%  Anxiety 27.1% |
| Wańkowicz (2020) | Poland | HCWs | 441 | 52.2% | 40.47 (4.93) | Depression, Anxiety | GAD-7, PHQ-9 | Depression 70.7%  Anxiety 64.6% |
| Wong (2020) | China | GP | 3555 | 78.2% | ≥18 | Anxiety | STAI-6 | Anxiety 52.1% |
| Xiamonig (2020) | China | HCWs | 8817 | 78% | ≤31 | Depression, Anxiety | PHQ-9, GAD-7 | Depression 30.2%  Anxiety 20.7% |
| Xiao (2020) | China | HCWs | 958 | 67.2% | ≥18 | Depression, Anxiety, Stress | PSS-14, HAD | Depression 58%  Anxiety 54.2%  Stress 55.1% |
| Xing (2020) | China | HCWs | 548 | 72.08% | ≤25-≥45 | Depression, Anxiety | SCL-90 | Depression 29.7%  Anxiety 34.1% |
| Xiong (2020) | China | HCWs | 223 | 97.3% | ≤25-55 | Depression, Anxiety | PHQ-9, GAD-7 | Depression 40.8%  Anxiety 26.4% |
| Youssef (2020) | Egypt | HCWs | 540 | 45.6% | 20-70 | Depression, Anxiety, Stress | DASS-21 | Depression 41.8%  Anxiety 26.5%  Stress 25% |
| Zandifar (2020) | Iran | HCWs | 869 | 71.23% | ≥18 | Depression, Anxiety, Stress | DASS-21 | Depression 41.7%  Anxiety 51.2%  Stress 33.9% |
| Zhan (2020) | China | HCWs | 2667 | 96.96% | 30 (26.35) | Depression, Anxiety, Stress | PHQ-9, CPSS | Depression 54.7%  Anxiety 39.8%  Stress 62% |
| Zhang (2020) | Iran | HCWs | 304 | 58.6% | 35.1 (9.1) | Depression, Anxiety | PHQ-9 | Depressionb20.6%  Anxiety 28% |
| Zhang (2020) | China | GP | 123768 | 29.44% | 30.3 (6.4) | Depression, Anxiety | SAS, SDS | Depression 22.8%  Anxiety 3.4% |
| Zhang (2020) | China | GP | 3237 | 47.1% | ≥18-83 | Depression, Anxiety | PHQ-9, GAD-7 | Depression 21.7%  Anxiety 19.5% |
| Zhou (2020) | China | GP  HCWs | GP 1099  HCWs 606 | - | *GP*  29.23 (10.33)  *HCWs*  35.77 (8.13) | Depression, Anxiety | PHQ-9, GAD-7 | *GP:*  Depression 47.6%  Anxiety 33.8%  *HCWs:*  Depression 57.6%  Anxiety 45.4% |
| Zhu (2020) | China | HCWs | 165 | 83% | 34.16 (8.06) | Depression, Anxiety | SAS, SDS | Depression 45.6%  Anxiety 11.4% |
| Zhu (2020) | China | HCWs | 453 | 94.9% | ≥18 | Anxiety | SAS | Anxiety 40.8% |
| Zhu (2020) | China | HCWs | 5062 | 85% | ≥18 | Depression, Anxiety, Stress | IES-R, PHQ-9, GAD-7 | Depression 13.5%  Anxiety 24.1%  Stress 29.8% |

GP = general population; HCWs = healthcare workers; PHQ= patient health questionnaire; GAD= general anxiety disorder; SAS = statistical anxiety scale; SDS= self–rating depression scale; IES-R = impact of event scale–revised; CPSS = Chinese version of the perceived stress scale; DASS-21 = depression, anxiety, and stress scale; SCL-90 = symptom checklist; PSS = perceived stress scale; HADS = hospital anxiety and depression scale; STAI= state–trait anxiety inventory; EEP-10 = “escala de estrés percibido”; CES-D = center for epidemiologic studies–depression; WHO-5 = world health organization well-being index; DMHWB = danish mental health and well-being survey; ASDS = acute stress disorder scale; PC-PTSD = primary care posttraumatic stress disorder; PSS = perceived stress scale; EASE = acute stress of health professionals caring COVID-19 scale; IESR = impact of event scale–revised; BDI = beck depression inventory; STAY-Y1 = state–trait anxiety inventory; HAMA= Hamilton anxiety scale; HAMD = Hamilton depression scale; PEHWSRPSHID = psychometric evaluation of healthcare workers' stress related to caring for patients with a highly infectious disease; ODSIS = overall depression severity and impairment scale; OASIS = overall anxiety severity and impairment scale; WEMWBS = Warwick-Edinburgh mental well-being scale

**Table S4.**

*Quality Assessment (JBI) of the Studies Included in the Meta-analysis*

| First author (year) | 1 | 2 | 3 | 4 | 5 | 6 | 7 | 8 | 9 | TOTAL |
| --- | --- | --- | --- | --- | --- | --- | --- | --- | --- | --- |
| Agberotimi (2020) | N | Y | Y | Y | Y | Y | Y | Y | U | 7 |
| Al Bana (2020) | N | Y | Y | Y | Y | Y | Y | Y | Y | 8 |
| Alan (2020) | N | U | Y | Y | Y | Y | Y | Y | U | 6 |
| AlAteeq (2020) | N | Y | Y | Y | Y | Y | Y | Y | N | 7 |
| Alkhamees (2020) | N | Y | Y | Y | Y | Y | Y | Y | N | 7 |
| Alshekaili (2020) | N | N | Y | Y | Y | Y | Y | Y | N | 6 |
| Alzueta (2020) | N | Y | Y | Y | Y | Y | Y | Y | U | 7 |
| Amin (2020) | N | Y | Y | Y | Y | Y | Y | Y | Y | 8 |
| An (2020) | N | Y | Y | Y | Y | Y | Y | Y | N | 7 |
| Amerio (2020) | N | N | U | Y | Y | Y | Y | Y | N | 5 |
| Antonijevic (2020) | N | Y | Y | Y | Y | Y | Y | Y | N | 7 |
| Arnetz (2020) | N | Y | Y | Y | Y | Y | Y | Y | N | 7 |
| Arshad (2020) | N | Y | Y | Y | Y | Y | Y | Y | Y | 8 |
| Awano (2020) | N | N | Y | N | Y | Y | Y | Y | N | 5 |
| Azoulay (2020) | N | Y | Y | N | Y | Y | N | Y | Y | 6 |
| Balsamo (2020) | N | Y | Y | Y | Y | Y | Y | Y | N | 7 |
| Barzilay (2020) | N | Y | Y | N | Y | Y | N | Y | N | 5 |
| Bäuerle (2020) | N | N | Y | Y | Y | Y | Y | Y | Y | 7 |
| Benke (2020) | N | Y | Y | Y | Y | Y | Y | Y | N | 7 |
| Bigalke (2020) | N | Y | Y | Y | Y | Y | Y | Y | Y | 8 |
| Blekas (2020) | N | N | Y | Y | Y | Y | Y | Y | N | 6 |
| Bobes-Bascarán (2020) | N | N | Y | Y | Y | Y | Y | Y | N | 6 |
| Bressington (2020) | N | U | Y | Y | Y | Y | Y | Y | N | 6 |
| Burhamah (2020) | N | Y | Y | Y | Y | Y | Y | Y | Y | 8 |
| Campos (2020) | N | Y | Y | Y | Y | Y | Y | Y | Y | 8 |
| Casagrande (2020) | N | Y | Y | N | Y | Y | N | Y | Y | 6 |
| Chatterjee (2020) | N | N | Y | Y | Y | Y | Y | Y | N | 6 |
| Chen (2020) | N | N | Y | Y | Y | Y | N | Y | Y | 6 |
| Cheng (2020) | N | N | Y | Y | Y | Y | Y | Y | N | 6 |
| Chew (2020) | N | N | Y | Y | Y | Y | Y | Y | N | 6 |
| Cunill (2020) | N | Y | Y | Y | Y | Y | Y | Y | Y | 8 |
| Czeisle (2020) | N | Y | Y | Y | Y | Y | Y | Y | Y | 8 |
| Dawson (2020) | N | Y | Y | Y | Y | Y | Y | Y | Y | 8 |
| Di Tella (2020) | N | Y | Y | Y | Y | Y | Y | Y | N | 7 |
| Elbay (2020) | N | U | Y | Y | Y | Y | Y | Y | N | 6 |
| Evanoff (2020) | N | N | Y | Y | Y | Y | Y | Y | N | 6 |
| Every-Palmer (2020) | N | N | Y | Y | Y | Y | Y | Y | Y | 7 |
| Fauzi (2020) | N | N | Y | Y | Y | Y | Y | Y | N | 6 |
| Fiorillo (2020) | N | N | Y | Y | Y | Y | Y | Y | N | 6 |
| Fisher (2020) | N | Y | N | Y | Y | Y | Y | Y | Y | 7 |
| Fitzpatrick (2020) | Y | N | Y | Y | Y | Y | Y | Y | N | 7 |
| Fu (2020) | N | Y | Y | N | Y | Y | Y | Y | N | 6 |
| Galindo-Vázquez (2020) | N | Y | Y | Y | Y | Y | Y | Y | N | 7 |
| Gallagher (2020) | N | Y | Y | Y | Y | Y | Y | Y | N | 7 |
| Giusti (2020) | N | Y | Y | Y | Y | Y | Y | Y | Y | 8 |
| Glowacks (2020) | N | Y | Y | Y | Y | Y | Y | Y | N | 7 |
| González-Sanguino (2020) | N | Y | Y | Y | Y | Y | Y | Y | N | 7 |
| Grover (2020) | N | Y | Y | Y | Y | Y | Y | Y | Y | 8 |
| Gualano (2020) | N | N | Y | Y | Y | Y | Y | Y | N | 6 |
| Günther-Bel (2020) | N | N | Y | Y | Y | Y | Y | Y | N | 6 |
| Guo (2020) | N | Y | Y | Y | Y | Y | Y | Y | Y | 8 |
| Gurvich (2020) | N | Y | Y | Y | Y | Y | Y | Y | U | 7 |
| Hasan (2020) | N | N | Y | Y | Y | Y | Y | Y | N | 6 |
| Hennein (2020) | N | Y | Y | Y | Y | Y | N | Y | N | 6 |
| Horesh (2020) | N | N | Y | Y | Y | Y | Y | Y | N | 6 |
| Hou (2020) | N | Y | Y | Y | Y | Y | Y | Y | N | 7 |
| Hu (2020) | N | N | Y | Y | Y | Y | Y | Y | N | 6 |
| Huang (2020) | N | N | Y | Y | Y | Y | Y | Y | Y | 7 |
| Huang (2020) | N | Y | Y | Y | Y | Y | Y | Y | N | 7 |
| Hyland (2020) | N | N | Y | Y | Y | Y | Y | Y | N | 6 |
| Jia (2020) | N | Y | N | N | Y | Y | Y | Y | N | 5 |
| Johnson (2020) | N | Y | N | N | Y | Y | Y | Y | N | 5 |
| Khanal (2020) | N | N | Y | Y | Y | Y | Y | Y | N | 6 |
| Korkmaz (2020) | N | U | Y | Y | Y | Y | Y | Y | N | 6 |
| Kuo (2020) | N | N | Y | Y | Y | Y | Y | Y | Y | 7 |
| Lai (2020) | N | Y | Y | Y | Y | Y | Y | Y | N | 7 |
| Lam (2020) | N | Y | Y | Y | Y | Y | Y | Y | N | 7 |
| Landi (2020) | N | N | Y | Y | Y | Y | Y | Y | Y | 7 |
| Lei (2020) | N | Y | Y | Y | Y | Y | Y | Y | N | 7 |
| Li (2020) | N | Y | Y | Y | Y | Y | Y | Y | N | 7 |
| Li (2020) | N | Y | Y | N | Y | Y | Y | Y | Y | 7 |
| Li (2020) | N | N | Y | Y | Y | Y | Y | Y | Y | 7 |
| Liang (2020) | N | N | Y | Y | Y | Y | Y | Y | Y | 7 |
| Liu (2020) | N | N | Y | Y | Y | Y | Y | Y | N | 6 |
| Liu (2020) | N | N | Y | Y | Y | Y | Y | Y | N | 6 |
| Liu (2020) | N | N | N | Y | Y | Y | N | Y | N | 4 |
| Lu (2020) | N | Y | Y | Y | Y | Y | Y | Y | Y | 8 |
| Lu (2020) | N | Y | Y | Y | Y | Y | Y | Y | Y | 8 |
| Luceño-Moreno (2020) | N | Y | Y | Y | Y | Y | Y | Y | Y | 8 |
| Ma (2020) | N | Y | Y | N | Y | Y | Y | Y | Y | 7 |
| Magnavita (2020) | N | N | Y | Y | Y | Y | Y | Y | Y | 7 |
| Margaretha (2020) | N | Y | Y | Y | Y | Y | Y | Y | Y | 8 |
| Massad (2020) | N | Y | Y | Y | Y | Y | Y | Y | N | 7 |
| Mazza (2020) | N | Y | Y | Y | Y | Y | Y | Y | N | 7 |
| Milgrom (2020) | N | N | Y | Y | Y | Y | Y | Y | N | 6 |
| Mira (2020) | N | N | Y | Y | Y | Y | Y | Y | N | 6 |
| Moghanibashi- Mansourieh (2020) | N | N | Y | N | Y | Y | Y | Y | N | 5 |
| Munk (2020) | N | N | Y | Y | Y | Y | Y | Y | N | 6 |
| Naser (2020) | N | N | Y | Y | Y | Y | Y | Y | N | 6 |
| Newby (2020) | N | Y | Y | Y | Y | Y | Y | Y | N | 7 |
| Ni (2020) | N | N | Y | Y | Y | Y | Y | Y | Y | 7 |
| Ozamiz-Etxebarria (2020) | N | Y | Y | Y | N | Y | Y | Y | N | 6 |
| Ozamiz-Etxebarria (2020) | N | Y | Y | N | Y | Y | Y | Y | Y | 7 |
| Özdin (2020) | N | Y | Y | Y | Y | Y | Y | Y | N | 7 |
| Pandey (2020) | N | N | Y | Y | Y | Y | Y | Y | N | 6 |
| Papandreou (2020) | N | Y | Y | Y | Y | Y | Y | Y | N | 7 |
| Park (2020) | N | N | Y | Y | N | Y | Y | Y | Y | 6 |
| Parlapani (2020) | N | N | Y | N | Y | Y | Y | Y | N | 5 |
| Passos (2020) | N | Y | Y | N | Y | Y | Y | Y | Y | 7 |
| Pérez-Cano (2020) | N | Y | Y | Y | Y | Y | Y | Y | N | 7 |
| Perveen (2020) | N | N | Y | Y | Y | Y | Y | Y | N | 6 |
| Pieh (2020) | N | N | Y | Y | Y | Y | Y | Y | N | 6 |
| Qian (2020) | N | Y | Y | N | Y | Y | Y | Y | N | 6 |
| Que (2020) | Y | Y | Y | Y | Y | Y | Y | Y | Y | 9 |
| Ran (2020) | N | Y | Y | Y | Y | Y | Y | Y | N | 7 |
| Ren (2020) | N | Y | Y | N | Y | Y | Y | Y | Y | 7 |
| Ren (2020) | N | Y | Y | Y | Y | Y | N | Y | N | 6 |
| Robb (2020) | N | Y | Y | Y | Y | Y | Y | Y | N | 7 |
| Rodríguez-Rey (2020) | N | N | Y | Y | Y | Y | Y | Y | N | 6 |
| Rossi (2020) | N | N | Y | Y | Y | Y | Y | Y | N | 6 |
| Rossi (2020) | N | Y | N | Y | Y | Y | Y | Y | N | 6 |
| Şahin (2020) | N | Y | Y | Y | Y | Y | N | Y | N | 6 |
| Sandín (2020) | U | N | Y | Y | Y | Y | Y | Y | N | 6 |
| Shatla (2020) | N | Y | Y | Y | Y | Y | Y | Y | N | 7 |
| Shechter (2020) | N | Y | Y | Y | Y | Y | Y | Y | N | 7 |
| Sherman (2020) | N | Y | Y | Y | Y | Y | Y | Y | Y | 8 |
| Shevlin (2020) | N | Y | Y | Y | Y | Y | Y | Y | Y | 8 |
| Shi (2020) | Y | U | Y | Y | Y | Y | Y | Y | Y | 8 |
| Shrestha (2020) | N | Y | Y | Y | Y | Y | Y | Y | Y | 8 |
| Si (2020) | N | Y | Y | Y | Y | Y | Y | Y | N | 7 |
| Solomou (2020) | N | Y | Y | Y | Y | Y | Y | Y | Y | 8 |
| Sonderskov (2020) | N | Y | Y | N | N | Y | Y | Y | N | 5 |
| Song (2020) | N | Y | N | N | Y | Y | N | Y | N | 4 |
| Song (2020) | N | Y | Y | Y | Y | Y | Y | Y | N | 7 |
| Stanton (2020) | N | Y | Y | Y | Y | Y | Y | Y | N | 7 |
| Stylianou (2020) | N | N | Y | Y | Y | Y | Y | Y | N | 6 |
| Su (2020) | N | N | Y | Y | Y | Y | Y | Y | U | 6 |
| Tang (2020) | N | Y | N | N | Y | Y | Y | Y | Y | 6 |
| Tee (2020) | Y | Y | Y | Y | Y | Y | Y | Y | U | 8 |
| Temsah (2020) | N | Y | Y | N | Y | Y | Y | Y | N | 6 |
| Teng (2020) | N | Y | Y | Y | Y | Y | Y | Y | Y | 8 |
| Tian (2020) | N | Y | Y | Y | Y | Y | Y | Y | N | 7 |
| Torales (2020) | N | Y | Y | Y | Y | Y | Y | Y | Y | 8 |
| Tu (2020) | N | Y | N | Y | Y | Y | Y | Y | N | 6 |
| Twenge (2020) | N | Y | Y | Y | Y | Y | Y | Y | Y | 8 |
| Ustun (2020) | U | N | Y | Y | Y | Y | Y | Y | N | 6 |
| Verma (2020) | N | Y | Y | Y | Y | Y | Y | Y | N | 7 |
| Wang (2020) | N | Y | Y | Y | Y | Y | Y | Y | Y | 8 |
| Wang (2020) | N | U | Y | Y | Y | Y | Y | Y | Y | 7 |
| Wang (2020) | N | Y | Y | N | Y | Y | N | Y | N | 5 |
| Wang (2020) | N | Y | Y | N | Y | Y | Y | Y | Y | 7 |
| Wang (2020) | N | N | Y | Y | Y | Y | Y | Y | Y | 7 |
| Wańkowicz (2020) | N | N | Y | Y | Y | Y | Y | Y | N | 6 |
| Wong (2020) | N | Y | Y | Y | Y | Y | Y | Y | N | 7 |
| Xiamonig (2020) | N | Y | Y | Y | Y | Y | Y | Y | Y | 8 |
| Xiao (2020) | N | Y | Y | Y | Y | Y | N | Y | N | 6 |
| Xing (2020) | N | Y | Y | Y | Y | Y | Y | Y | N | 7 |
| Xiong (2020) | N | Y | Y | Y | Y | Y | Y | Y | Y | 8 |
| Youssef (2020) | N | Y | Y | Y | Y | Y | Y | Y | N | 7 |
| Zandifar (2020) | N | N | Y | Y | Y | Y | Y | U | N | 5 |
| Zhan (2020) | N | Y | Y | Y | Y | Y | Y | Y | Y | 8 |
| Zhang (2020) | N | N | Y | Y | Y | Y | Y | N | N | 5 |
| Zhang (2020) | N | Y | Y | Y | N | Y | Y | Y | N | 6 |
| Zhang (2020) | N | Y | N | Y | Y | Y | Y | Y | Y | 7 |
| Zhou (2020) | N | Y | Y | Y | Y | Y | Y | Y | N | 7 |
| Zhu (2020) | N | Y | Y | Y | Y | Y | Y | Y | Y | 8 |
| Zhu (2020) | N | Y | Y | Y | Y | Y | Y | Y | N | 7 |
| Zhu (2020) | N | N | Y | Y | Y | Y | Y | Y | Y | 7 |

**References**

Agberotimi, S. F., Akinsola, O. S., Oguntayo, R., & Olaseni, A. O. (2020). Interactions between socioeconomic status and mental health outcomes in the Nigerian context amid COVID-19 pandemic: A comparative study. *Frontiers in Psychology*, *11*, 1–11. https://doi.org/10.3389/fpsyg.2020.559819

Al Banna, M. H., Sayeed, A., Kundu, S., Christopher, E., Hasan, M. T., Begum, M. R., Kormoker, T., Dola, S. T. I., Hassan, M. M., Chowdhury, S., & Khan, M. S. I. (2020). The impact of the COVID-19 pandemic on the mental health of the adult population in Bangladesh: a nationwide cross-sectional study. *International Journal of Environmental Health Research*, 1–12. https://doi.org/10.1080/09603123.2020.1802409

Alan, H., Eskin Bacaksiz, F., Tiryaki Sen, H., Taskiran Eskici, G., Gumus, E., & Harmanci Seren, A. K. (2020). “I’m a hero, but…”: An evaluation of depression, anxiety, and stress levels of frontline healthcare professionals during COVID-19 pandemic in Turkey. *Perspectives in Psychiatric Care*, *57*(3), 1126–1136. https://doi.org/10.1111/ppc.12666

AlAteeq, D. A., Aljhani, S., Althiyabi, I., & Majzoub, S. (2020). Mental health among healthcare providers during coronavirus disease (COVID-19) outbreak in Saudi Arabia. *Journal of Infection and Public Health*, *13*(10), 1432–1437. https://doi.org/10.1016/j.jiph.2020.08.013

Alkhamees, A. A., Alrashed, S. A., Alzunaydi, A. A., Almohimeed, A. S., & Aljohani, M. S. (2020). The psychological impact of COVID-19 pandemic on the general population of Saudi Arabia. *Comprehensive Psychiatry*, *102*, 1–9. https://doi.org/10.1016/j.comppsych.2020.152192

Alshekaili, M., Hassan, W., Al Said, N., Al Sulaimani, F., Jayapal, S. K., Al-Mawali, A., Chan, M. F., Mahadevan, S., & Al-Adawi, S. (2020). Factors associated with mental health outcomes across healthcare settings in Oman during COVID-19: Frontline versus non-frontline healthcare workers. *BMJ Open*, *10*(10). https://doi.org/10.1136/bmjopen-2020-042030

Alzueta, E., Perrin, P., Baker, F. C., Caffarra, S., Ramos-Usuga, D., Yuksel, D., & Arango-Lasprilla, J. C. (2020). How the COVID-19 pandemic has changed our lives: A study of psychological correlates across 59 countries. *Journal of Clinical Psychology*, *77*(3), 556–570. https://doi.org/10.1002/jclp.23082

Amerio, A., Bianchi, D., Santi, F., Costantini, L., Odone, A., Signorelli, C., Costanza, A., Serafini, G., Amore, M., & Aguglia, A. (2020). Covid-19 pandemic impact on mental health: A web-based cross-sectional survey on a sample of Italian general practitioners. *Acta Biomedica*, *91*(2), 83–88. https://doi.org/10.23750/abm.v91i2.9619

Amin, F., Sharif, S., Saeed, R., Durrani, N., & Jilani, D. (2020). COVID-19 pandemic- knowledge, perception, anxiety and depression among frontline doctors of Pakistan. *BMC Psychiatry*, *20*(1). https://doi.org/10.1186/S12888-020-02864-X

An, Y., Yang, Y., Wang, A., Li, Y., Zhang, Q., Cheung, T., Ungvari, G. S., Qin, M. Z., An, F. R., & Xiang, Y. T. (2020). Prevalence of depression and its impact on quality of life among frontline nurses in emergency departments during the COVID-19 outbreak. *Journal of Affective Disorders*, *276*, 312–315. https://doi.org/10.1016/j.jad.2020.06.047

Antonijevic, J., Binic, I., Zikic, O., Manojlovic, S., Tosic-Golubovic, S., & Popovic, N. (2020). Mental health of medical personnel during the COVID-19 pandemic. *Brain and Behavior*, *10*(12), 2–9. https://doi.org/10.1002/brb3.1881

Arnetz, J. E., Goetz, C. M., Sudan, S., Arble, E., Janisse, J., & Arnetz, B. B. (2020). Personal protective equipment and mental health symptoms among nurses during the COVID-19 pandemic. *Journal of Occupational and Environmental Medicine*, *62*(11), 892–897. https://doi.org/10.1097/JOM.0000000000001999

Arshad, A. R., & Islam, F. (2020). COVID-19 and anxiety amongst doctors: A Pakistani perspective. *Journal of the College of Physicians and Surgeons Pakistan*, *30*(2), 106–109. https://doi.org/10.29271/JCPSP.2020.SUPP2.106

Awano, N., Oyama, N., Akiyama, K., Inomata, M., Kuse, N., Tone, M., Takada, K., Muto, Y., Fujimoto, K., Akagi, Y., Mawatari, M., Ueda, A., Kawakami, J., Komatsu, J., & Izumo, T. (2020). Anxiety, depression, and resilience of healthcare workers in Japan during the coronavirus disease 2019 outbreak. *Internal Medicine*, *59*(21), 2693–2699. https://doi.org/10.2169/internalmedicine.5694-20

Azoulay, E., De Waele, J., Ferrer, R., Staudinger, T., Borkowska, M., Povoa, P., Iliopoulou, K., Artigas, A., Schaller, S. J., Hari, M. S., Pellegrini, M., Darmon, M., Kesecioglu, J., & Cecconi, M. (2020). Symptoms of burnout in intensive care unit specialists facing the COVID-19 outbreak. *Annals of Intensive Care*, *10*(110), 1–8. https://doi.org/10.1186/s13613-020-00722-3

Balsamo, M., & Carlucci, L. (2020). Italians on the age of COVID-19: The self-reported depressive symptoms through web-based survey. *Frontiers in Psychology*, *11*. https://doi.org/10.3389/fpsyg.2020.569276

Barzilay, R., Moore, T. M., Greenberg, D. M., DiDomenico, G. E., Brown, L. A., White, L. K., Gur, R. C., & Gur, R. E. (2020). Resilience, COVID-19-related stress, anxiety and depression during the pandemic in a large population enriched for healthcare providers. *Translational Psychiatry*, *10*(1). https://doi.org/10.1038/s41398-020-00982-4

Bäuerle, A., Steinbach, J., Schweda, A., Beckord, J., Hetkamp, M., Weismüller, B., Kohler, H., Musche, V., Dörrie, N., Teufel, M., & Skoda, E. M. (2020). Mental health burden of the COVID-19 outbreak in Germany: Predictors of mental health impairment. *Journal of Primary Care and Community Health*, *11*. https://doi.org/10.1177/2150132720953682

Benke, C., Autenrieth, L. K., Asselmann, E., & Pané-Farré, C. A. (2020). Lockdown, quarantine measures, and social distancing: Associations with depression, anxiety and distress at the beginning of the COVID-19 pandemic among adults from Germany. *Psychiatry Research*, *293*. https://doi.org/10.1016/j.psychres.2020.113462

Bigalke, J. A., Greenlund, I. M., & Carter, J. R. (2020). Sex differences in self-report anxiety and sleep quality during COVID-19 stay-at-home orders. *Biology of Sex Differences*, *11*(1). https://doi.org/10.1186/s13293-020-00333-4

Blekas, A., Voitsidis, P., Athanasiadou, M., Parlapani, E., Chatzigeorgiou, A. F., Skoupra, M., Syngelakis, M., Holeva, V., & Diakogiannis, I. (2020). COVID-19: PTSD symptoms in greek health care professionals. *Psychological Trauma: Theory, Research, Practice, and Policy*, *12*(7), 812–819. https://doi.org/10.1037/tra0000914

Bobes-Bascarán, T., Sáiz, P. A., Velasco, A., Martínez-Cao, C., Pedrosa, C., Portilla, A., de la Fuente-Tomas, L., García-Alvarez, L., García-Portilla, M. P., & Bobes, J. (2020). Early psychological correlates associated with COVID-19 in a Spanish older adult sample. *American Journal of Geriatric Psychiatry*, *28*(12), 1287–1298. https://doi.org/10.1016/j.jagp.2020.09.005

Bressington, D. T., Cheung, T. C. C., Lam, S. C., Suen, L. K. P., Fong, T. K. H., Ho, H. S. W., & Xiang, Y. T. (2020). Association between depression, health beliefs, and face mask use during the COVID-19 pandemic. *Frontiers in Psychiatry*, *11*. https://doi.org/10.3389/fpsyt.2020.571179

Burhamah, W., AlKhayyat, A., Oroszlányová, M., AlKenane, A., Almansouri, A., Behbehani, M., Karimi, N., Jafar, H., & AlSuwaidan, M. (2020). The psychological burden of the COVID-19 pandemic and associated lockdown measures: Experience from 4000 participants. *Journal of Affective Disorders*, *277*, 977–985. https://doi.org/10.1016/j.jad.2020.09.014

Campos, J. A. D. B., Martins, B. G., Campos, L. A., Marôco, J., Saadiq, R. A., & Ruano, R. (2020). Early psychological impact of the COVID-19 pandemic in Brazil: A national survey. *Journal of Clinical Medicine*, *9*(9), 1–14. https://doi.org/10.3390/jcm9092976

Casagrande, M., Favieri, F., Tambelli, R., & Forte, G. (2020). The enemy who sealed the world: effects quarantine due to the COVID-19 on sleep quality, anxiety, and psychological distress in the Italian population. *Sleep Medicine*, *75*, 12–20. https://doi.org/10.1016/j.sleep.2020.05.011

Chatterjee, S. S., Bhattacharyya, R., Bhattacharyya, S., Gupta, S., Das, S., & Banerjee, B. B. (2020). Attitude, practice, behavior, and mental health impact of COVID-19 on doctors. *Indian Journal of Psychiatry*, *62*(3), 257–265. https://doi.org/10.4103/psychiatry.IndianJPsychiatry_333_20

Chen, B., Qing-xian, L., Zhang, H., Zhu, J., Yang, X., Wu, Y., Xiong, J., Fu, L., Hua, W., & Chen, Z. (2020). The psychological impact of COVID-19 outbreak on medical staff and the general public. *Current Psychology*. https://doi.org/10.1007/s12144-020-01109-0

Cheng, F. F., Zhan, S. H., Xie, A. W., Cai, S. Z., Hui, L., Kong, X. X., Tian, J. M., & Yan, W. H. (2020). Anxiety in Chinese pediatric medical staff during the outbreak of Coronavirus Disease 2019: A cross-sectional study. *Translational Pediatrics*, *9*(3), 231–236. https://doi.org/10.21037/TP.2020.04.02

Chew, N. W. S., Lee, G. K. H., Tan, B. Y. Q., Jing, M., Goh, Y., Ngiam, N. J. H., Yeo, L. L. L., Ahmad, A., Ahmed Khan, F., Napolean Shanmugam, G., Sharma, A. K., Komalkumar, R. N., Meenakshi, P. V., Shah, K., Patel, B., Chan, B. P. L., Sunny, S., Chandra, B., Ong, J. J. Y., … Sharma, V. K. (2020). A multinational, multicentre study on the psychological outcomes and associated physical symptoms amongst healthcare workers during COVID-19 outbreak. *Brain, Behavior, and Immunity*, *88*, 559–565. https://doi.org/10.1016/j.bbi.2020.04.049

Cunill, M., Aymerich, M., Serdà, B. C., & Patiño-Masó, J. (2020). The impact of COVID-19 on Spanish health professionals: A description of physical and psychological effects. *International Journal of Mental Health Promotion*, *22*(3), 185–198. https://doi.org/10.32604/IJMHP.2020.011615

Czeisler, M. É., Lane, R. I., Petrosky, E., Wiley, J. F., Christensen, A., Njai, R., Weaver, M. ., Robbins, R., Facer-Childs, E. R., Barger, L. K., Czeisler, C. A., Howard, M. E., & Rajaratnam, S. M. (2020). Mental health, substance use, and suicidal ideation during the COVID-19 pandemic—United States, June 24–30, 2020. *Morbidity and Mortality Weekly Report*, *69*(32), 1049–1057. https://doi.org/10.15585/mmwr.mm6932a1

Dawson, D. L., & Golijani-Moghaddam, N. (2020). COVID-19: Psychological flexibility, coping, mental health, and wellbeing in the UK during the pandemic. *Journal of Contextual Behavioral Science*, *17*, 126–134. https://doi.org/10.1016/j.jcbs.2020.07.010

Di Tella, M., Romeo, A., Benfante, A., & Castelli, L. (2020). Mental health of healthcare workers during the COVID-19 pandemic in Italy. *Journal of Evaluation in Clinical Practice*, *26*(6), 1583–1587. https://doi.org/10.1111/jep.13444

Elbay, R. Y., Kurtulmuş, A., Arpacıoğlu, S., & Karadere, E. (2020). Depression, anxiety, stress levels of physicians and associated factors in Covid-19 pandemics. *Psychiatry Research*, *290*, 113–130. https://doi.org/10.1016/j.psychres.2020.113130

Evanoff, B. A., Strickland, J. R., Dale, A. M., Hayibor, L., Page, E., Duncan, J. G., Kannampallil, T., & Gray, D. L. (2020). Work-related and personal factors associated with mental well-being during the COVID-19 response: Survey of health care and other workers. *Journal of Medical Internet Research*, *22*(8). https://doi.org/10.2196/21366

Every-Palmer, S., Jenkins, M., Gendall, P., Hoek, J., Beaglehole, B., Bell, C., Williman, J., Rapsey, C., & Stanley, J. (2020). Psychological distress, anxiety, family violence, suicidality, and wellbeing in New Zealand during the COVID-19 lockdown: A cross-sectional study. *PLoS ONE*, *15*(11 November). https://doi.org/10.1371/journal.pone.0241658

Fauzi, M. F. M., Yusoff, H. M., Robat, R. M., Saruan, N. A. M., Ismail, K. I., & Haris, A. F. M. (2020). Doctors’ mental health in the midst of covid-19 pandemic: The roles of work demands and recovery experiences. *International Journal of Environmental Research and Public Health*, *17*(19), 1–16. https://doi.org/10.3390/ijerph17197340

Fiorillo, A., Sampogna, G., Giallonardo, V., Del Vecchio, V., Luciano, M., Albert, U., Carmassi, C., Carrà, G., Cirulli, F., Dell’Osso, B., Nanni, M. G., Pompili, M., Sani, G., Tortorella, A., & Volpe, U. (2020). Effects of the lockdown on the mental health of the general population during the COVID-19 pandemic in Italy: Results from the COMET collaborative network. *European Psychiatry*, *63*(1). https://doi.org/10.1192/j.eurpsy.2020.89

Fisher, J. R., Tran, T. D., Hammarberg, K., Sastry, J., Nguyen, H., Rowe, H., Popplestone, S., Stocker, R., Stubber, C., & Kirkman, M. (2020). Mental health of people in Australia in the first month of COVID-19 restrictions: a national survey. *Medical Journal of Australia*, *213*(10), 458–464. https://doi.org/10.5694/mja2.50831

Fitzpatrick, K. M., Harris, C., & Drawve, G. (2020). Living in the midst of fear: Depressive symptomatology among US adults during the COVID-19 pandemic. *Depression and Anxiety*, *37*(10), 957–964. https://doi.org/10.1002/da.23080

Fu, W., Wang, C., Zou, L., Guo, Y., Lu, Z., Yan, S., & Mao, J. (2020). Psychological health, sleep quality, and coping styles to stress facing the COVID-19 in Wuhan, China. *Translational Psychiatry*, *10*(1). https://doi.org/10.1038/s41398-020-00913-3

Galindo-Vázquez, O., Ramírez-Orozco, M., Costas-Muñiz, R., Mendoza-Contreras, L. A., Calderillo-Ruíz, G., & Meneses-García, A. (2020). Symptoms of anxiety, depression and self-care behaviors during the COVID-19 pandemic in the general population. *Gaceta Medica de Mexico*, *156*(4), 298–305. https://doi.org/10.24875/GMM.20000266

Gallagher, M. W., Zvolensky, M. J., Long, L. J., Rogers, A. H., & Garey, L. (2020). The Impact of Covid-19 Experiences and Associated Stress on Anxiety, Depression, and Functional Impairment in American Adults. *Cognitive Therapy and Research*, *44*(6), 1043–1051. https://doi.org/10.1007/s10608-020-10143-y

Giusti, E. M., Pedroli, E., D’Aniello, G. E., Stramba Badiale, C., Pietrabissa, G., Manna, C., Stramba Badiale, M., Riva, G., Castelnuovo, G., & Molinari, E. (2020). The psychological impact of the COVID-19 outbreak on health professionals: A cross-sectional study. *Frontiers in Psychology*, *11*, 1–9. https://doi.org/10.3389/fpsyg.2020.01684

Glowacz, F., & Schmits, E. (2020). Psychological distress during the COVID-19 lockdown: The young adults most at risk. *Psychiatry Research*, *293*, 1–4. https://doi.org/10.1016/j.psychres.2020.113486

González-Sanguino, C., Ausín, B., Castellanos, M. Á., Saiz, J., López-Gómez, A., Ugidos, C., & Muñoz, M. (2020). Mental health consequences during the initial stage of the 2020 Coronavirus pandemic (COVID-19) in Spain. *Brain, Behavior, and Immunity*, *87*, 172–176. https://doi.org/10.1016/j.bbi.2020.05.040

Grover, S., Sahoo, S., Mehra, A., Avasthi, A., Tripathi, A., Subramanyan, A., Pattojoshi, A., Rao, G. P., Saha, G., Mishra, K. K., Chakraborty, K., Rao, N. P., Vaishnav, M., Singh, O. P., Dalal, P. K., Chadda, R. ., Gupta, R., Gautam, S., Sarkar, S., … Janardran Reddy, Y. C. (2020). Psychological impact of COVID-19 lockdown: An online survey from India: Few concerns. *Indian Journal of Psychiatry*, *62*(4), 354–362. https://doi.org/10.4103/psychiatry.IndianJPsychiatry_427_20

Gualano, M. R., Lo Moro, G., Voglino, G., Bert, F., & Siliquini, R. (2020). Effects of COVID-19 lockdown on mental health and sleep disturbances in Italy. *International Journal of Environmental Research and Public Health*, *17*(13), 1–13. https://doi.org/10.3390/ijerph17134779

Günther-Bel, C., Vilaregut, A., Carratala, E., Torras-Garat, S., & Pérez-Testor, C. (2020). A mixed-method study of individual, couple, and parental functioning during the state-regulated COVID-19 lockdown in Spain. *Family Process*, *59*(3), 1060–1079. https://doi.org/10.1111/famp.12585

Guo, Y., Cheng, C., Zeng, Y., Li, Y., Zhu, M., Yang, W., Xu, H., Li, X., Leng, J., Monroe-Wise, A., & Wu, S. (2020). Mental health disorders and associated risk factors in quarantined adults during the COVID-19 outbreak in China: Cross-sectional study. *Journal of Medical Internet Research*, *22*(8). https://doi.org/10.2196/20328

Gurvich, C., Thomas, N., Thomas, E. H. X., Hudaib, A. R., Sood, L., Fabiatos, K., Sutton, K., Isaacs, A., Arunogiri, S., Sharp, G., & Kulkarni, J. (2020). Coping styles and mental health in response to societal changes during the COVID-19 pandemic. *International Journal of Social Psychiatry*, *67*(5), 540–549. https://doi.org/10.1177/0020764020961790

Hasan, S. R., Hamid, Z., Jawaid, M. T., & Ali, R. K. (2020). Anxiety among doctors during COVID-19 pandemic in secondary and tertiary care hospitals. *Pakistan Journal of Medical Sciences*, *36*(6), 1360–1365. https://doi.org/10.12669/pjms.36.6.3113

Hennein, R., & Lowe, S. (2020). A hybrid inductive-abductive analysis of health workers’ experiences and wellbeing during the COVID-19 pandemic in the United States. *PLoS ONE*, *15*(10), 1–21. https://doi.org/10.1371/journal.pone.0240646

Horesh, D., Kapel Lev-Ari, R., & Hasson-Ohayon, I. (2020). Risk factors for psychological distress during the COVID-19 pandemic in Israel: Loneliness, age, gender, and health status play an important role. *British Journal of Health Psychology*, *25*(4), 925–933. https://doi.org/10.1111/bjhp.12455

Hou, F., Bi, F., Jiao, R., Luo, D., & Song, K. (2020). Gender differences of depression and anxiety among social media users during the COVID-19 outbreak in China:a cross-sectional study. *BMC Public Health*, *20*(1), 1–11. https://doi.org/10.1186/s12889-020-09738-7

Hu, D., Kong, Y., Li, W., Han, Q., Zhang, X., Zhu, L. X., Wan, S. W., Liu, Z., Shen, Q., Yang, J., He, H. G., & Zhu, J. (2020). Frontline nurses’ burnout, anxiety, depression, and fear statuses and their associated factors during the COVID-19 outbreak in Wuhan, China: A large-scale cross-sectional study. *EClinicalMedicine*, *24*, 1–10. https://doi.org/10.1016/j.eclinm.2020.100424

Huang, Y., & Zhao, N. (2020a). Chinese mental health burden during the COVID-19 pandemic. *Asian Journal of Psychiatry*, *51*, 1–3. https://doi.org/10.1016/j.ajp.2020.102052

Huang, Y., & Zhao, N. (2020b). Generalized anxiety disorder, depressive symptoms and sleep quality during COVID-19 outbreak in China: a web-based cross-sectional survey. *Psychiatry Research*, *288*, 1–6. https://doi.org/10.1016/j.psychres.2020.112954

Hyland, P., Shevlin, M., McBride, O., Murphy, J., Karatzias, T., Bentall, R. P., Martinez, A., & Vallières, F. (2020). Anxiety and depression in the Republic of Ireland during the COVID-19 pandemic. *Acta Psychiatrica Scandinavica*, *142*(3), 249–256. https://doi.org/10.1111/acps.13219

Jia, R., Ayling, K., Chalder, T., Massey, A., Broadbent, E., Coupland, C., & Vedhara, K. (2020). Mental health in the UK during the COVID-19 pandemic: cross-sectional analyses from a community cohort study. *BMJ Open*, *10*(9), 1–14. https://doi.org/10.1136/bmjopen-2020-040620

Johnson, S. U., Ebrahimi, O. V., & Hoffart, A. (2020). PTSD symptoms among health workers and public service providers during the COVID-19 outbreak. *PLoS ONE*, *15*(10), 1–13. https://doi.org/10.1371/journal.pone.0241032

Khanal, P., Devkota, N., Dahal, M., Paudel, K., & Joshi, D. (2020). Mental health impacts among health workers during COVID-19 in a low resource setting: A cross-sectional survey from Nepal. *Globalization and Health*, *16*(1). https://doi.org/10.1186/s12992-020-00621-z

Korkmaz, S., Kazgan, A., Çekiç, S., Tartar, A. S., Balcı, H. N., & Atmaca, M. (2020). The anxiety levels, quality of sleep and life and problem-solving skills in healthcare workers employed in COVID-19 services. *Journal of Clinical Neuroscience*, *80*, 131–136. https://doi.org/10.1016/j.jocn.2020.07.073

Kuo, F. L., Yang, P. H., Hsu, H. T., Su, C. Y., Chen, C. H., Yeh, I. J., Wu, Y. H., & Chen, L. C. (2020). Survey on perceived work stress and its influencing factors among hospital staff during the COVID-19 pandemic in Taiwan. *Kaohsiung Journal of Medical Sciences*, *36*(11), 944–952. https://doi.org/10.1002/kjm2.12294

Lai, J., Ma, S., Wang, Y., Cai, Z., Hu, J., Wei, N., Wu, J., Du, H., Chen, T., Li, R., Tan, H., Kang, L., Yao, L., Huang, M., Wang, H., Wang, G., Liu, Z., & Hu, S. (2020). Factors associated with mental health outcomes among health care workers exposed to coronavirus disease 2019. *JAMA Network Open*, *3*(3), 203976. https://doi.org/10.1001/jamanetworkopen.2020.3976

Lam, S. C., Arora, T., Grey, I., Suen, L. K. P., Huang, E. Y. zhi, Li, D., & Lam, K. B. H. (2020). Perceived risk and protection from infection and depressive symptoms among healthcare workers in Mainland China and Hong Kong during COVID-19. *Frontiers in Psychiatry*, *11*, 1–7. https://doi.org/10.3389/fpsyt.2020.00686

Landi, G., Pakenham, K. I., Boccolini, G., Grandi, S., & Tossani, E. (2020). Health anxiety and mental health outcome during COVID-19 lockdown in Italy: The mediating and moderating roles of psychological flexibility. *Frontiers in Psychology*, *11*, 1–14. https://doi.org/10.3389/fpsyg.2020.02195

Lei, L., Huang, X., Zhang, S., Yang, J., Yang, L., & Xu, M. (2020). Comparison of prevalence and associated factors of anxiety and depression among people affected by versus people unaffected by quarantine during the COVID-19 epidemic in southwestern China. *Medical Science Monitor*, *26*. https://doi.org/10.12659/MSM.924609

Li, R., Chen, Y., Lv, J., Liu, L., Zong, S., Li, H., & Li, H. (2020). Anxiety and related factors in frontline clinical nurses fighting COVID-19 in Wuhan. *Medicine*, *99*(30), 1–5. https://doi.org/10.1097/MD.0000000000021413

Li, Q., Chen, J., Xu, G., Zhao, J., Yu. X., Wang, S., Liu, L., & Liu, F. (2020). ﻿Psychological status of healthcare workers during the Covid-19 pandemic Outbreak: A Cross-Sectional Survey Study in Guangdong, China. *Frontiers in Public Health, 8*, 1–9*.* <https://doi.org/10.3389/fpubh.2020.562885>

Li, X., Lu, P., Hu, L., Huang, T., & Lu, L. (2020). Factors associated with mental health results among workers with income losses exposed to COVID‐19 in China. *International Journal of Environmental Research and Public Health*, *17*(15), 1–11. https://doi.org/10.3390/ijerph17155627

Liang, Y., Wu, K., Zhou, Y., Huang, X., Zhou, Y., & Liu, Z. (2020). Mental health in frontline medical workers during the 2019 novel coronavirus disease epidemic in China: A comparison with the general population. *International Journal of Environmental Research and Public Health*, *17*(18), 1–12. https://doi.org/10.3390/ijerph17186550

Liu, C. H., Zhang, E., Wong, G. T. F., Hyun, S., & Hahm, H. “Chris.” (2020). Factors associated with depression, anxiety, and PTSD symptomatology during the COVID-19 pandemic: Clinical implications for U.S. young adult mental health. *Psychiatry Research*, *290*. https://doi.org/10.1016/j.psychres.2020.113172

Liu, C. Y., Yang, Y. Z., Zhang, X. M., Xu, X., Dou, Q. L., Zhang, W. W., & Cheng, A. S. K. (2020). The prevalence and influencing factors in anxiety in medical workers fighting COVID-19 in China: A cross-sectional survey. *Epidemiology and Infection*, *148*, 1–7. https://doi.org/10.1017/S0950268820001107

Liu, Y., Wang, L., Chen, L., Zhang, X., Bao, L., & Shi, Y. (2020). Mental health status of paediatric medical workers in China during the COVID-19 outbreak. *Frontiers in Psychiatry*, *11*, 1–7. https://doi.org/10.3389/fpsyt.2020.00702

Lu, P., Li, X., Lu, L., & Zhang, Y. (2020). The psychological states of people after Wuhan eased the lockdown. *PLoS ONE*, *15*(11), 1–12. https://doi.org/10.1371/journal.pone.0241173

Lu, W., Wang, H., Lin, Y., & Li, L. (2020). Psychological status of medical workforce during the COVID-19 pandemic: A cross-sectional study. *Psychiatry Research*, *288*, 1–5. https://doi.org/10.1016/j.psychres.2020.112936

Luceño-Moreno, L., Talavera-Velasco, B., García-Albuerne, Y., & Martín-García, J. (2020). Symptoms of posttraumatic stress, anxiety, depression, levels of resilience and burnout in spanish health personnel during the COVID-19 pandemic. *International Journal of Environmental Research and Public Health*, *17*(15), 1–29. https://doi.org/10.3390/ijerph17155514

Ma, Y., Rosenheck, R., & He, H. (2020). Psychological stress among health care professionals during the 2019 novel coronavirus disease Outbreak: Cases from online consulting customers. *Intensive and Critical Care Nursing*, *61*. https://doi.org/10.1016/j.iccn.2020.102905

Magnavita, N., Tripepi, G., & Di Prinzio, R. R. (2020). Symptoms in health care workers during the covid-19 epidemic. A cross-sectional survey. *International Journal of Environmental Research and Public Health*, *17*(14), 1–15. https://doi.org/10.3390/ijerph17145218

Margaretha, S. E. P. M., Effendy, C., Kusnanto, H., & Hasinuddin, M. (2020). Determinants psychological distress of indonesian health care providers during COVID-19 pandemic. *Systematic Reviews in Pharmacy*, *11*(6), 1052–1059. https://doi.org/10.31838/srp.2020.6.150

Massad, I., Al-Taher, R., Massad, F., Al-Sabbagh, M. Q., Haddad, M., & Abufaraj, M. (2020). The impact of the covid-19 pandemic on mental health: Early quarantine-related anxiety and its correlates among jordanians. *Eastern Mediterranean Health Journal*, *26*(10), 1165–1172. https://doi.org/10.26719/emhj.20.115

Mazza, C., Ricci, E., Biondi, S., Colasanti, M., Ferracuti, S., Napoli, C., & Roma, P. (2020). A nationwide survey of psychological distress among italian people during the covid-19 pandemic: Immediate psychological responses and associated factors. *International Journal of Environmental Research and Public Health*, *17*(9). https://doi.org/10.3390/ijerph17093165

Milgrom, Y., Tal, Y., & Finestone, A. S. (2020). Comparison of hospital worker anxiety in COVID-19 treating and non-treating hospitals in the same city during the COVID-19 pandemic. *Israel Journal of Health Policy Research*, *9*(1). https://doi.org/10.1186/s13584-020-00413-1

Mira, J. J., Carrillo, I., Guilabert, M., Mula, A., Martin-Delgado, J., Pérez-Jover, M. V., Vicente, M. A., & Fernández, C. (2020). Acute stress of the healthcare workforce during the COVID-19 pandemic evolution: A cross-sectional study in Spain. *BMJ Open*, *10*(11). https://doi.org/10.1136/bmjopen-2020-042555

Moghanibashi-Mansourieh, A. (2020). Assessing the anxiety level of Iranian general population during COVID-19 outbreak. *Asian Journal of Psychiatry*, *51*. https://doi.org/10.1016/j.ajp.2020.102076

Munk, A. J. L., Schmid, N. M., Alexander, N., Henkel, K., & Hennig, J. (2020). Covid-19-Beyond virology: Potentials for maintaining mental health during lockdown. *PLoS ONE*, *15*(8). https://doi.org/10.1371/journal.pone.0236688

Naser, A. Y., Dahmash, E. Z., Al-Rousan, R., Alwafi, H., Alrawashdeh, H. M., Ghoul, I., Abidine, A., Bokhary, M. A., AL-Hadithi, H. T., Ali, D., Abuthawabeh, R., Abdelwahab, G. M., Alhartani, Y. J., Al Muhaisen, H., Dagash, A., & Alyami, H. S. (2020). Mental health status of the general population, healthcare professionals, and university students during 2019 coronavirus disease outbreak in Jordan: A cross-sectional study. *Brain and Behavior*, *10*(8), 1–13. https://doi.org/10.1002/brb3.1730

Newby, J. M., O’Moore, K., Tang, S., Christensen, H., & Faasse, K. (2020). Acute mental health responses during the COVID-19 pandemic in Australia. *PLoS ONE*, *15*(7), 1–21. https://doi.org/10.1371/journal.pone.0236562

Ni, M. Y., Yang, L., Leung, C. M. C., Li, N., Yao, X. I., Wang, Y., Leung, G. M., Cowling, B. J., & Liao, Q. (2020). Mental health, risk factors, and social media use during the COVID-19 epidemic and cordon sanitaire among the community and health professionals in wuhan, China: Cross-sectional survey. *JMIR Mental Health*, *7*(5). https://doi.org/10.2196/19009

Ozamiz-Etxebarria, N., Dosil-Santamaria, M., Picaza-Gorrochategui, M., & Idoiaga-Mondragon, N. (2020). Stress, anxiety, and depression levels in the initial stage of the COVID-19 outbreak in a population sample in the northern Spain. *Cadernos de Saude Publica*, *36*(4). https://doi.org/10.1590/0102-311X00054020

Ozamiz-Etxebarria, N., Idoiaga Mondragon, N., Dosil Santamaría, M., & Picaza Gorrotxategi, M. (2020). Psychological symptoms during the two stages of lockdown in response to the COVID-19 outbreak: An investigation in a sample of citizens in northern Spain. *Frontiers in Psychology*, *11*, 1–9. https://doi.org/10.3389/fpsyg.2020.02116

Özdin, S., & Bayrak Özdin, Ş. (2020). Levels and predictors of anxiety, depression and health anxiety during COVID-19 pandemic in Turkish society: The importance of gender. *International Journal of Social Psychiatry*, *66*(5), 504–511. https://doi.org/10.1177/0020764020927051

Pandey, D., Bansal, S., Goyal, S., Garg, A., Sethi, N., Pothiyill, D. I., Sreelakshmi, E. S., Sayyad, M. G., & Sethi, R. (2020). Psychological impact of mass quarantine on population during pandemics-The COVID-19 Lock-Down (COLD) study. *PLoS ONE*, *15*(10), 1–10. https://doi.org/10.1371/journal.pone.0240501

Papandreou, C., Arija, V., Aretouli, E., Tsilidis, K. K., & Bulló, M. (2020). Comparing eating behaviours, and symptoms of depression and anxiety between Spain and Greece during the COVID-19 outbreak: Cross-sectional analysis of two different confinement strategies. *European Eating Disorders Review*, *28*(6), 836–846. https://doi.org/10.1002/erv.2772

Park, S. Y., Kim, B., Jung, D. S., Jung, S. I., Oh, W. S., Kim, S. W., Peck, K. R., & Chang, H. H. (2020). Psychological distress among infectious disease physicians during the response to the COVID-19 outbreak in the Republic of Korea. *BMC public health, 20*(1), ﻿1811. https://doi.org/10.1186/s12889-020-09886-w

Parlapani, E., Holeva, V., Voitsidis, P., Blekas, A., Gliatas, I., Porfyri, G. N., Golemis, A., Papadopoulou, K., Dimitriadou, A., Chatzigeorgiou, A. F., Bairachtari, V., Patsiala, S., Skoupra, M., Papigkioti, K., Kafetzopoulou, C., & Diakogiannis, I. (2020). Psychological and behavioral responses to the COVID-19 pandemic in Greece. *Frontiers in Psychiatry*, *11*, 1–17. https://doi.org/10.3389/fpsyt.2020.00821

Passos, L., Prazeres, F., Teixeira, A., & Martins, C. (2020). Impact on mental health due to covid-19 pandemic: Cross-sectional study in portugal and brazil. *International Journal of Environmental Research and Public Health*, *17*(18), 1–13. https://doi.org/10.3390/ijerph17186794

Pérez-Cano, H. J., Moreno-Murguía, M. B., Morales-López, O., Crow-Buchanan, O., English, J. A., Lozano-Alcázar, J., & Somilleda-Ventura, S. A. (2020). Anxiety, depression, and stress in response to the coronavirus disease-19 pandemic. *Cirugia y Cirujanos (English Edition)*, *88*(5), 562–568. https://doi.org/10.24875/CIRU.20000561

Perveen, A., Hamzah, H. B., Othamn, A., & Ramlee, F. (2020). Prevalence of anxiety, stress, depression among malaysian adults during COVID-19 pandemic movement control order. *Indian Journal of Community Health*, *32*(3), 579–581. https://doi.org/10.47203/IJCH.2020.v32i03.020

Pieh, C., Budimir, S., & Probst, T. (2020). The effect of age, gender, income, work, and physical activity on mental health during coronavirus disease (COVID-19) lockdown in Austria. *Journal of Psychosomatic Research*, *136*. https://doi.org/10.1016/j.jpsychores.2020.110186

Qian, M., Wu, Q., Wu, P., Hou, Z., Liang, Y., Cowling, B. J., & Yu, H. (2020). Anxiety levels, precautionary behaviours and public perceptions during the early phase of the COVID-19 outbreak in China: A population-based cross-sectional survey. *BMJ Open*, *10*(10), 1–10. https://doi.org/10.1136/bmjopen-2020-040910

Que, J., Shi, L., Deng, J., Liu, J., Zhang, L., Wu, S., Gong, Y., Huang, W., Yuan, K., Yan, W., Sun, Y., Ran, M., Bao, Y., & Lu, L. (2020). Psychological impact of the covid-19 pandemic on healthcare workers: A cross-sectional study in China. *General Psychiatry*, *33*(3), 1–12. https://doi.org/10.1136/gpsych-2020-100259

Ran, L., Wang, W., Ai, M., Kong, Y., Chen, J., & Kuang, L. (2020). Psychological resilience, depression, anxiety, and somatization symptoms in response to COVID-19: A study of the general population in China at the peak of its epidemic. *Social Science and Medicine*, *262*, 1–6. https://doi.org/10.1016/j.socscimed.2020.113261

Ren, Y., Qian, W., Li, Z., Liu, Z., Zhou, Y., Wang, R., Qi, L., Yang, J., Song, X., Zeng, L., & Zhang, X. (2020). Public mental health under the long-term influence of COVID-19 in China: Geographical and temporal distribution. *Journal of Affective Disorders*, *277*, 893–900. https://doi.org/10.1016/j.jad.2020.08.045

Ren, Z., Zhou, Y., & Liu, Y. (2020). The psychological burden experienced by Chinese citizens during the COVID-19 outbreak: prevalence and determinants. *BMC Public Health*, *20*(1). https://doi.org/10.1186/s12889-020-09723-0

Robb, C. E., de Jager, C. A., Ahmadi-Abhari, S., Giannakopoulou, P., Udeh-Momoh, C., McKeand, J., Price, G., Car, J., Majeed, A., Ward, H., & Middleton, L. (2020). Associations of social Iiolation with anxiety and depression during the early COVID-19 pandemic: A survey of older adults in London, UK. *Frontiers in Psychiatry*, *11*, 1–12. https://doi.org/10.3389/fpsyt.2020.591120

Rodríguez-Rey, R., Garrido-Hernansaiz, H., & Collado, S. (2020). Psychological impact and associated factors during the initial stage of the coronavirus (COVID-19) pandemic among the general population in Spain. *Frontiers in Psychology*, *11*, 1–23. https://doi.org/10.3389/fpsyg.2020.01540

Rossi, R., Socci, V., Pacitti, F., Di Lorenzo, G., Di Marco, A., Siracusano, A., & Rossi, A. (2020). Mental health outcomes among frontline and second-line health care workers during the coronavirus disease 2019 (COVID-19) pandemic in Italy. In *JAMA Network Open*, *3*(5), 1–4. https://doi.org/10.1001/jamanetworkopen.2020.10185

Rossi, R., Socci, V., Talevi, D., Mensi, S., Niolu, C., Pacitti, F., Di Marco, A., Rossi, A., Siracusano, A., & Di Lorenzo, G. (2020). COVID-19 pandemic and lockdown measures impact on mental health among the general population in Italy. *Frontiers in Psychiatry*, *11*, 1–6. https://doi.org/10.3389/fpsyt.2020.00790

Şahin, M. K., Aker, S., Şahin, G., & Karabekiroğlu, A. (2020). Prevalence of depression, anxiety, distress and insomnia and related factors in healthcare workers during COVID-19 pandemic in Turkey. *Journal of Community Health*, *45*(6), 1168–1177. https://doi.org/10.1007/s10900-020-00921-w

Sandín, B., Valiente, R. M., García-Escalera, J., & Chorot, P. (2020). Psychological impact of the COVID-19 pandemic: Negative and positive effects in Spanish people during the mandatory national quarantine. *Revista de Psicopatologia y Psicologia Clinica*, *25*(1), 1–22. https://doi.org/10.5944/RPPC.27569

Shatla, M. M., Khafagy, A. A., Bulkhi, A. A., & Aljahdali, I. A. (2020). Public concerns and mental health changes related to the COVID-19 pandemic lockdown in Saudi Arabia. *Clinical Laboratory*, *6*(10), 2125–2132. https://doi.org/10.7754/Clin.Lab.2020.200614

Shechter, A., Diaz, F., Moise, N., Anstey, D. E., Ye, S., Agarwal, S., Birk, J. L., Brodie, D., Cannone, D. E., Chang, B., Claassen, J., Cornelius, T., Dong, M., Derby, L., Givens, R. C., Hochman, B., Homma, S., Kronish, I. M., ..., & Abdalla, M. (2020). Psychological distress, coping behaviors, and preferences for support among New York healthcare workers during the COVID-19 pandemic. *General Hospital Psychiatry*, *66*, 1–8.

Sherman, A. C., Williams, M. L., Amick, B. C., Hudson, T. J., & Messias, E. L. (2020). Mental health outcomes associated with the COVID-19 pandemic: Prevalence and risk factors in a southern US state. *Psychiatry Research*, *293*, 1–8. https://doi.org/10.1016/j.psychres.2020.113476

Shevlin, M., McBride, O., Murphy, J., Miller, J. G., Hartman, T. K., Levita, L., Mason, L., Martinez, A. P., McKay, R., Stocks, T. V. A., Bennett, K. M., Hyland, P., Karatzias, T., & Bentall, R. P. (2020). Anxiety, depression, traumatic stress and COVID-19-related anxiety in the UK general population during the COVID-19 pandemic. *BJPsych Open*, *6*(6), 1–9. https://doi.org/10.1192/bjo.2020.109

Shi, L., Lu, Z. A., Que, J. Y., Huang, X. L., Liu, L., Ran, M. S., Gong, Y.-M., Yuan, K., Yan, W., Sun, Y.-K., Shi, J., Bao, Y.-P., & Lu, L. (2020). Prevalence of and risk factors associated with mental health symptoms among the general population in China during the coronavirus disease 2019 pandemic. *JAMA Network Open*, *3*(7), 1–16. https://doi.org/10.1001/jamanetworkopen.2020.14053

Shrestha, C., Ghimire, C., Acharya, S., Prabhat, K. C., Singh, S., & Sharma, P. (2020). Mental wellbeing during the lockdown period following the COVID-19 pandemic in Nepal: A descriptive cross-sectional study. *Journal of the Nepal Medical Association*, *58*(230), 744–750. https://doi.org/10.31729/jnma.5498

Si, M. Y., Su, X. Y., Jiang, Y., Wang, W. J., Gu, X. F., Ma, L., Li, J., Zhang, S. K., Ren, Z. F., Ren, R., Liu, Y. L., & Qiao, Y. L. (2020). Psychological impact of COVID-19 on medical care workers in China. *Infectious Diseases of Poverty*, *9*(1), 1–13. https://doi.org/10.1186/s40249-020-00724-0

Solomou, I., & Constantinidou, F. (2020). Prevalence and predictors of anxiety and depression symptoms during the COVID-19 pandemic and compliance with precautionary measures: Age and sex matter. *International Journal of Environmental Research and Public Health*, *17*(14), 1–19. https://doi.org/10.3390/ijerph17144924

Sønderskov, K. M., Dinesen, P. T., Santini, Z. I., & Østergaard, S. D. (2020). The depressive state of Denmark during the COVID-19 pandemic. *Acta Neuropsychiatrica*, *32*(4), 226–228. https://doi.org/10.1017/neu.2020.15

Song, L., Wang, Y., Li, Z., Yang, Y., & Li, H. (2020). Mental health and work attitudes among people resuming work during the COVID-19 pandemic: A cross-sectional study in China. *International Journal of Environmental Research and Public Health*, *17*(14), 1–15. https://doi.org/10.3390/ijerph17145059

Song, X., Fu, W., Liu, X., Luo, Z., Wang, R., Zhou, N., Yan, S., & Lv, C. (2020). Mental health status of medical staffin emergency departments during theCoronavirus disease 2019 epidemic in China. *Brain, Behavior, and Immunity*, *88*, 60–65. https://doi.org/10.11817/j.issn.1672-7347.2020.200070

Stanton, R., To, Q. G., Khalesi, S., Williams, S. L., Alley, S. J., Thwaite, T. L., Fenning, A. S., & Vandelanotte, C. (2020). Depression, anxiety and stress during COVID-19: Associations with changes in physical activity, sleep, tobacco and alcohol use in Australian adults. *International Journal of Environmental Research and Public Health*, *17*(11), 1–13. https://doi.org/10.3390/ijerph17114065

Stylianou, N., Samouti, G., & Samoutis, G. (2020). Mental health disorders during the COVID-19 outbreak in Cyprus. *Journal of Medicine and Life*, *13*(3), 300–305. https://doi.org/10.25122/jml-2020-0114

Su, J., Chen, X., Yang, N., Sun, M., & Zhou, L. (2020). Proximity to people with COVID-19 and anxiety among community residents during the epidemic in Guangzhou, China. *BJPsych Open*, *6*(4), 1–3. https://doi.org/10.1192/bjo.2020.59

Temsah, M. H., Al-Sohime, F., Alamro, N., Al-Eyadhy, A., Al-Hasan, K., Jamal, A., Al-Maglouth, I., Aljamaan, F., Al Amri, M., Barry, M., Al-Subaie, S., & Somily, A. M. (2020). The psychological impact of COVID-19 pandemic on health care workers in a MERS-CoV endemic country. *J Infect Public Health, 13*(6), 877-882. https://doi.org/10.1016/j.jiph.2020.05.021

Tang, F., Liang, J., Zhang, H., Kelifa, M. M., He, Q., & Wang, P. (2020). COVID-19 related depression and anxiety among quarantined respondents. *Psychology and Health*, *36*(2), 164–178. https://doi.org/10.1080/08870446.2020.1782410

Tee, M. L., Tee, C. A., Anlacan, J. P., Aligam, K. J. G., Reyes, P. W. C., Kuruchittham, V., & Ho, R. C. (2020). Psychological impact of COVID-19 pandemic in the Philippines. *Journal of Affective Disorders*, *277*, 379–391. https://doi.org/10.1016/j.jad.2020.08.043

Teng, Z., Wei, Z., Qiu, Y., Tan, Y., Chen, J., Tang, H., Wu, H., Wu, R., & Huang, J. (2020). Psychological status and fatigue of frontline staff two months after the COVID-19 pandemic outbreak in China: A cross-sectional study. *Journal of Affective Disorders*, *275*, 247–252. https://doi.org/10.1016/j.jad.2020.06.032

Tian, F., Li, H., Tian, S., Yang, J., Shao, J., & Tian, C. (2020). Psychological symptoms of ordinary Chinese citizens based on SCL-90 during the level I emergency response to COVID-19. *Psychiatry Research*, *288*, 1–9. https://doi.org/10.1016/j.psychres.2020.112992

Torales, J., Ríos-González, C., Barrios, I., O’Higgins, M., González, I., García, O., Castaldelli-Maia, J. M., & Ventriglio, A. (2020). Self-perceived stress during the quarantine of COVID-19 pandemic in Paraguay: An exploratory survey. *Frontiers in Psychiatry*, *11*, 1–6. https://doi.org/10.3389/fpsyt.2020.558691

Tu, Z. H., He, J. W., & Zhou, N. (2020). Sleep quality and mood symptoms in conscripted frontline nurse in Wuhan, China during COVID-19 outbreak: A cross-sectional study. *Medicine, 99*(26). https://doi.org/ ﻿10.1097/MD.0000000000020769

Twenge, J. M., & Joiner, T. E. (2020). U.S. Census Bureau-assessed prevalence of anxiety and depressive symptoms in 2019 and during the 2020 COVID-19 pandemic. *Depression and Anxiety*, *37*(10), 954–956. https://doi.org/10.1002/da.23077

Ustun, G. (2021). Determining depression and related factors in a society affected by COVID-19 pandemic. *International Journal of Social Psychiatry*, *67*(1), 54–63. https://doi.org/10.1177/0020764020938807

Verma, S., & Mishra, A. (2020). Depression, anxiety, and stress and socio-demographic correlates among general Indian public during COVID-19. *International Journal of Social Psychiatry, 66*(8)*, ﻿*756-762. <https://doi.org/﻿10.1177/0020764020934508>

Wang, Y., Ma, S., Yang, C., Cai, Z., Hu, S., Zhang, B., Tang, S., Bai, H., Guo, X., Wu, J., Du, H., Kang, L., Tan, H., Li, R., Yao, L., Wang, G., & Liu, Z. (2020). Acute psychological effects of Coronavirus Disease 2019 outbreak among healthcare workers in China: a cross-sectional study. *Translational Psychiatry, 10*(1)*.* <https://doi.org/10.1038/s41398-020-01031-w>

Wang, C., Pan, R., Wan, X., Tan, Y., Xu, L., Ho, S. C., & Ho, R. C. (2020). Immediate Psychological Responses and Associated Factors during the Initial Stage of the 2019 Coronavirus Disease (COVID-19) Epidemic among the General Population in China. *International Journal of Environmental Research and Public Health, 17*(5)*.* <https://doi.org/﻿10.3390/ijerph17051729>

Wang, W., Song, W., Xia, Z., He. Y., Tang, L., Hou, J., & Lei, S. (2020). Sleep disturbance and psychological profiles of medical staff and non-medical staff during the early outbreak of COVID-19 in Hubei province, China. *Frontiers in Psychiatry, 11.* <https://doi.org/﻿10.3389/fpsyt.2020.00733>

Wang, S., Zhang, Y., Ding, W., Meng, Y., Hu, H., Liu, Z., Zeng, X., Wang, M. (2020). Psychological distress and sleep problems when people are under interpersonal isolation during an epidemic: A nationwide multicenter cross-sectional study. *European Psychiatry, 63*(1). <https://doi.org/10.1192/j.eurpsy.2020.78>

Wang, L. Q., Zhang, M., Liu, G. M., Nan, S. Y., Li, T., Xu, L., Xue, Y., Zhang, M., Wang, L., Qu, Y. D., & Liu, F. (2020). Psychological impact of coronavirus disease (2019) (COVID-19) epidemic on medical staff in different posts in China: A multicenter study. *Journal of Psychiatric Research, 129,* ﻿198-205. <https://doi.org/10.1016/j.jpsychires.2020.07.008>

Wańkowicz, P., Szylińska, A., & Rotter, I. (2020). Assessment of mental health factors among health professionals depending on their contact with covid-19 patients. *International Journal of Environmental Research and Public Health,17*(16), 1-8. <https://doi.org/﻿10.3390/ijerph17165849>

Wong, L. P., Hung, C. C., Alias, H., & Lee, T. S. H. (2020). Anxiety symptoms and preventive measures during the COVID-19 outbreak in Taiwan. *BMC Psychiatry, 20*(1). <https://doi.org/10.1186/s12888-020-02786-8>

Xiamonig, X., Ming, A., Su, H., Wo, W., Jiannmei, C., Qi, Z., Hua, H., Xuemei, L., Lixia, W., Jun, C., Lei, S., Zhen, L., Lian, D., Jing, L., Handan, Y., Haitanng, Q., Xiaoting, H., Xiaorong, C., Ran, C., Qinghua, L., Xinyu, Z., Jian, T., Jing, T., Guanghua, J., Zhiqin, H., Nkundimana, B., & Li, K. (2020). The psychological status of 8817 hospital workers during COVID-19 Epidemic: A cross-sectional study in Chongqing. *Journal of Affective Disorders, 276, ﻿*555-561. https://doi.org/ ﻿10.1016/j.jad.2020.07.092

Xiao, X., Zhu, X., Fu, S., Hu, Y., Li, X., & Xiao, J. (2020). Psychological impact of healthcare workers in China during COVID-19 pneumonia epidemic: A multi-center cross-sectional survey investigation. *Journal of Affective Disorders, 274,* ﻿405-410. <https://doi.org/10.1016/j.jad.2020.05.081>

Xing, J., Sun, N., Xu, J., Gens, S., & Li, Y. (2020). Study of the mental health status of medical personnel dealing with new coronavirus pneumonia. *PLoS ONE, 15*(5). <https://doi.org/﻿10.1371/journal.pone.0233145>

Xiong, H., Yi, S., & Lin, Y. (2020). The psychological status and self-efficacy of nurses during COVID-19 outbreak: A cross-sectional survey. *Inquiry (United States), 57.* <https://doi.org/10.1177/0046958020957114>

Zhan, Y. X., Zhao, S. Y., Yuan, J., Liu, H., Liu, Y. F., Gui, L. L., Zheng, H., Zhou, Y. M., Qiu, L. H., Chen, J. H., Yu, J. H., & Li, S. Y. (2020). Prevalence and influencing factors on fatigue of first-line nurses combating with COVID-19 in China: A descriptive cross-sectional study. *Current Medical Science, 40*(4), ﻿625-635. https://doi.org/10.1007/s11596-020-2226-9

Zhang, Y., Wang, S., Ding, W., Meng, Y., Hu, H., Liu, Z., Zeng, X., Guan, Y., & Wang, M. (2020). Status and influential factors of anxiety depression and insomnia symptoms in the work resumption period of COVID-19 epidemic: A multicenter cross-sectional study. *Journal of Psychosomatic Research, 138.* <https://doi.org/﻿10.1016/j.jpsychores.2020.110253>

Zhang, X. R., Huang, Q. M., Wang, X. M., Cheng, X., Li, Z. H., Wang, Z. H., Zhong, W. F., Liu, D., Shen, D., Chen, P. L., Song, W. Q., Wu, X. B., Yang, X., & Mao, C. (2020). Prevalence of anxiety and depression symptoms, and association with epidemic-related factors during the epidemic period of COVID-19 among 123,768 workers in China: A large cross-sectional study. *Journal of Affective Disorders, 277*, ﻿495-502. <https://doi.org/﻿10.1016/j.jad.2020.08.041>

Zhou, Y., Wang, W., Sunn, Y., Qian, W., Liu, Z., Wang, R., Qi, L., Yang, J., Song, X., Zhou, X., Zeng, L., Liu, T., Li, Z., & Zhang, X. (2020). The prevalence and risk factors of psychological disturbances of frontline medical staff in china under the COVID-19 epidemic: Workload should be concerned. *Journal of Affective Disorders, 277*, ﻿510-514. <https://doi.org/﻿10.1016/j.jad.2020.08.059>

Zhu, J., Sun, L., Zhang, L., Wang, H., Fan, A., Yang, B., Li, W., & Xiao, S. (2020). Prevalence and influencing factors of anxiety and depression symptoms in the first-line medical staff fighting against COVID-19 in Gansu. *Frontiers in Psychiatry, 11.*

Zhu, W., Wei, Y., Meng, X., & Li, J. (2020). The mediation effects of coping style on the relationship between social support and anxiety in Chinese medical staff during COVID-19. *BMC Health Services Research, 20*(1). <https://doi.org/﻿0.1186/s12913-020-05871-6>

Zhu, Z., Xu, S., Wang, H., Liu, Z., Wu, J., Li, G., Miao, J., Zhang, C., Yang, Y., Sun, W., Zhu, S., Fan, Y., Chen, Y., Hu, J., Liu, J., & Wang, W. (2020). COVID-19 in Wuhan: Sociodemographic characteristics and hospital support measures associated with the immediate psychological impact on healthcare workers. *E Clinical Medicine, 24.* https://doi.org/﻿10.1016/j.eclinm.2020.100443
